# Patterns of joint involvement in juvenile idiopathic arthritis and prediction of disease course: A prospective study with multilayer non-negative matrix factorization

**DOI:** 10.1371/journal.pmed.1002750

**Published:** 2019-02-26

**Authors:** Simon W. M. Eng, Florence A. Aeschlimann, Mira van Veenendaal, Roberta A. Berard, Alan M. Rosenberg, Quaid Morris, Rae S. M. Yeung, on behalf of the ReACCh-Out Research Consortium

**Affiliations:** 1 Division of Rheumatology, Department of Paediatrics, The Hospital for Sick Children (SickKids), Toronto, Ontario, Canada; 2 Department of Immunology, University of Toronto, Toronto, Ontario, Canada; 3 Vector Institute, Toronto, Ontario, Canada; 4 Institute of Medical Science, University of Toronto, Toronto, Ontario, Canada; 5 Division of Rheumatology, Children’s Hospital, London Health Sciences Centre; 6 Department of Pediatrics, Schulich School of Medicine & Dentistry, Western University, London, Ontario, Canada; 7 Department of Pediatrics, University of Saskatchewan, Saskatoon, Saskatchewan, Canada; 8 The Donnelly Centre for Cellular and Biomolecular Research, University of Toronto, Toronto, Ontario, Canada; 9 Department of Molecular Genetics, University of Toronto, Toronto, Ontario, Canada; 10 Department of Computer Science, University of Toronto, Toronto, Ontario, Canada; 11 Ontario Institute for Cancer Research, Toronto, Ontario, Canada; Edinburgh University, UNITED KINGDOM

## Abstract

**Background:**

Joint inflammation is the common feature underlying juvenile idiopathic arthritis (JIA). Clinicians recognize patterns of joint involvement currently not part of the International League of Associations for Rheumatology (ILAR) classification. Using unsupervised machine learning, we sought to uncover data-driven joint patterns that predict clinical phenotype and disease trajectories.

**Methods and findings:**

We analyzed prospectively collected clinical data, including joint involvement using a standard 71-joint homunculus, for 640 discovery patients with newly diagnosed JIA enrolled in a Canada-wide study who were followed serially for five years, treatment-naïve except for nonsteroidal anti-inflammatory drugs (NSAIDs) and diagnosed within one year of symptom onset. Twenty-one patients had systemic arthritis, 300 oligoarthritis, 125 rheumatoid factor (RF)-negative polyarthritis, 16 RF-positive polyarthritis, 37 psoriatic arthritis, 78 enthesitis-related arthritis (ERA), and 63 undifferentiated arthritis. At diagnosis, we observed global hierarchical groups of co-involved joints.

To characterize these patterns, we developed sparse multilayer non-negative matrix factorization (NMF). Model selection by internal bi-cross-validation identified seven joint patterns at presentation, to which all 640 discovery patients were assigned: pelvic girdle (57 patients), fingers (25), wrists (114), toes (48), ankles (106), knees (283), and indistinct (7). Patterns were distinct from clinical subtypes (*P* < 0.001 by χ^2^ test) and reproducible through external data set validation on a 119-patient, prospectively collected independent validation cohort (reconstruction accuracy *Q*^2^ = 0.55 for patterns; 0.35 for groups).

Some patients matched multiple patterns. To determine whether their disease outcomes differed, we further subdivided the 640 discovery patients into three subgroups by degree of localization—the percentage of their active joints aligning with their assigned pattern: localized (≥90%; 359 patients), partially localized (60%–90%; 124), or extended (<60%; 157). Localized patients more often maintained their baseline patterns (*P* < 0.05 for five groups by permutation test) than nonlocalized patients (*P* < 0.05 for three groups by permutation test) over a five-year follow-up period.

We modelled time to zero joints in the discovery cohort using a multivariate Cox proportional hazards model considering joint pattern, degree of localization, and ILAR subtype. Despite receiving more intense treatment, 50% of nonlocalized patients had zero joints at one year compared to six months for localized patients. Overall, localized patients required less time to reach zero joints (partial: *P* = 0.0018 versus localized by log-rank test; extended: *P* = 0.0057).

Potential limitations include the requirement for patients to be treatment naïve (except NSAIDs), which may skew the patient cohorts towards milder disease, and the validation cohort size precluded multivariate analyses of disease trajectories.

**Conclusions:**

Multilayer NMF identified patterns of joint involvement that predicted disease trajectory in children with arthritis. Our hierarchical unsupervised approach identified a new clinical feature, degree of localization, which predicted outcomes in both cohorts. Detailed assessment of every joint is already part of every musculoskeletal exam for children with arthritis. Our study supports both the continued collection of detailed joint involvement and the inclusion of patterns and degrees of localization to stratify patients and inform treatment decisions. This will advance pediatric rheumatology from counting joints to realizing the potential of using data available from uncovering patterns of joint involvement.

## Abstract 2

Using unsupervised machine learning, Rae Yeung and colleagues uncover data-driven joint patterns that predict clinical phenotype and disease trajectories in JIA.

## Author summary

**Why was this study done?:**

- Juvenile idiopathic arthritis (JIA) is a heterogeneous childhood disease for which joint inflammation is the common denominator.
- The current classification system, the International League of Associations for Rheumatology (ILAR) subtypes, categorize patients according to the number of active joints (four or fewer versus five or more affected joints in the first six months of disease) with little evidence to support this cut-off.
- Grouping frequently co-involved joints into joint patterns may help to better classify patients and predict disease course.

**What did the researchers do and find?:**

- We analyzed baseline joint involvement data from 640 treatment-naïve patients from 16 Canadian centers participating in the Research in Arthritis in Canadian Children, Emphasizing Outcomes (ReACCh-Out) study between 2005 and 2010.
- We identified seven joint patterns that grouped frequently co-involved joints using multilayer non-negative matrix factorization (NMF), an unsupervised pattern recognition technique. We named these patterns as follows: pelvic girdle, fingers, wrists, toes, knees, ankles, and indistinct.
- These seven patterns described more homogenous groupings of joints than the ILAR subtypes and further stratified them.
- Throughout disease course, we found that patients with joint involvements largely overlapping their baseline patterns, i.e., those having localized involvement, continued to have the same active joints, whereas patients with nonlocalized involvement did not.
- Patients with localized joint involvement reached zero joint involvement faster than those with nonlocalized involvement.

**What do these findings mean?:**

- Patterns of joint involvement represent prognostic features that should be incorporated into a comprehensive JIA disease classification.
- Patients with nonlocalized joint involvement are at high risk of poor outcome.
- Grouping patients by joint pattern and degree of localization may help clinicians tailor treatments based on predicted disease trajectories.

## Introduction

Juvenile idiopathic arthritis (JIA) is the most common chronic inflammatory rheumatic disease in childhood, characterized by joint inflammation of unknown etiology lasting at least six weeks starting at <16 years of age. The International League of Associations for Rheumatology (ILAR) criteria classify JIA into seven subtypes [1].

Clinicians acknowledge that individual joints or groups of joints influence outcome. Distinct joint involvement patterns such as dactylitis, sacroiliac joint involvement, or tarsitis are clearly recognized in patients with JIA [1]. However, despite efforts to characterize distinct disease entities, these patterns remain heterogeneous in clinical presentation and consequently disease course and response to treatment. In addition, individual joints such as the hip, cervical spine, ankle, or wrist may be indicators of poor outcome [2–5] and have therefore been considered in the 2011 American College of Rheumatology (ACR) treatment recommendations as indicator joints for poor prognosis [6–8]. However, existing classification subtypes and treatment recommendations do not take affected joint patterns into account [1,6]. Moreover, these findings only apply to a subset of JIA patients and are based on a small number of joints [9,10]. A significant gap remains in our understanding of the relationship between patterns of joint involvement and disease outcome.

Systematically collected data from the Research in Arthritis in Canadian Children, Emphasizing Outcomes (ReACCh-Out) study have enabled us to address this knowledge gap. ReACCh-Out is a prospective inception cohort study of children with newly diagnosed JIA [11] that has rigorously collected detailed longitudinal clinical data, including information on joint inflammation. A standard 71-joint homunculus precludes the use of traditional statistical—or supervised learning—techniques to associate individual joints, let alone combinations of joints, to descriptors of outcome in an unbiased manner due to multiple hypothesis testing in the context of small patient numbers, a common challenge in rare diseases. To address this challenge, unsupervised learning can help identify a small number of patterns of arthritis in a principled, logical, and expressive manner, whose outcomes we can then explore. We can draw from previous successes applying these approaches in JIA analyzing clinical and biomarker data [12,13]. These patterns not only provide possible predictive features of outcome but also help to organize the patients into homologous groups that may underlie further disease research.

In this study, we pursued a data-driven strategy in a novel data domain, joint involvements, to identify two easily measurable clinical variables—joint pattern and degree of localization—in children with newly diagnosed JIA. We then determined the relationship of these measures to longitudinal outcomes, including pattern stability and response to treatment.

## Methods

Our analysis, outlined as a flow chart in S1 Fig, can be divided into the following phases: data collection and filtering, dimensionality reduction, clustering, and postclustering analysis. As indicated in our transparent reporting of a multivariable prediction model for individual prognosis or diagnosis (TRIPOD) checklist (S1 Checklist), our postclustering analysis plan was developed after we completed the clustering and observed that some patients defied identification with a single joint grouping. This observation prompted us to include a variable describing this in our multivariate prediction model.

### Study design

For the discovery cohort, patients enrolled in ReACCh-Out were included if they satisfied the classification criteria for any of the seven ILAR JIA subtypes [1]. Enrolled patients were included within one year of JIA diagnosis and were treatment naïve for medications except for nonsteroidal anti-inflammatory drugs (NSAIDs). ReACCh-Out is a prospective inception cohort of newly diagnosed patients with JIA recruited between 2005 and 2010 from 16 tertiary Canadian centers. These 16 centers included 14 academic and two community centers: Royal Jubilee Hospital, Victoria, British Columbia; BC Children’s Hospital, Vancouver, British Columbia; Penticton Regional Hospital, Penticton, British Columbia; University of Calgary, Calgary, Alberta; Alberta Health Services, Edmonton, Alberta; Royal University Hospital, Saskatoon, Saskatchewan; Health Sciences Centre, Winnipeg, Manitoba; The Hospital for Sick Children (SickKids), Toronto, Canada; Children’s Hospital of Eastern Ontario, Ottawa, Ontario; Montréal Children’s Hospital, Montréal, Québec; Université de Montréal, Montréal, Québec; Université de Sherbrooke, Sherbrooke, Québec; le Centre Hospitalier Universitaire de Québec, Québec, Québec; Santé et Services Sociaux, Québec, Québec; IWK Health Centre, Halifax, Nova Scotia; and Janeway Children’s Hospital and Rehabilitation Centre, St. John’s, Newfoundland [11].

The nonoverlapping independent validation cohort comprised prospectively collected patients with newly diagnosed chronic childhood arthritis and detailed information about joint involvement recruited between 1980 and 2007 from two tertiary Canadian sites: Royal University Hospital of Saskatoon, Saskatchewan and Health Sciences Centre of Winnipeg, Manitoba [14]. These sites were chosen because they had standardized collection of prospective data by the same personnel over the study timeframe. For this study, all patients were treatment naïve except for NSAIDs. Prior to the introduction of the ILAR classification criteria, patients in the validation cohort were identified as satisfying ACR criteria for juvenile rheumatoid arthritis (JRA).

Research ethics boards at each participating center approved the study protocols. Informed written consent for participation was obtained from parents, and informed consent or assent was obtained from patients as appropriate.

### Demographic, clinical, and laboratory data

For the discovery cohort, detailed demographic, clinical, and laboratory data were collected prospectively at study entry using standardized clinical reporting forms. Collected information included key features of the ILAR classification [1], the ACR pediatric core set measures of disease activity [15], standard laboratory markers, and physician assessments of global disease activity. All forms were subsequently validated. Musculoskeletal information for 71 joints as well as treatment regimens were serially collected at six-month intervals for the first 18 months and yearly thereafter for five years after study enrollment.

In the validation cohort, the features of the ILAR classification were collected as well as joint involvements at all subsequent clinical visits.

### Hardware and software

All analyses were conducted using R version 3.3.0 (Vienna, Austria) and Python version 3.6 and higher (Wilmington, Delaware, US) on Apple computers running Mac OS X 10.10 and higher, as well as at supercomputing facilities at the High Performance Facility at SickKids (Toronto, Ontario, Canada) and the Terrence Donnelly Centre for Cellular and Biomolecular Research at the University of Toronto (Toronto, Ontario, Canada).

### Joint co-involvements

Initially, joint co-involvements—which describe pairs of joints that are active together—were analyzed by calculating joint co-involvement frequencies and conditional joint co-involvement frequencies. A joint co-involvement frequency, *P*(*x*,*y*), for a reference joint *x* and a co-involved joint *y* is the proportion of patients having both joints involved. The conditional joint co-involvement frequencies, $P(y|x)=\frac{P(y,x)}{P(x)}$, are the fraction of all patients with *x* involved who also have *y* involved.

To investigate whether joint co-involvement frequencies were asymmetric, i.e., higher for joints on the same side of the body, we developed a measure of same-side versus opposite-side skew in the co-involvement frequencies computed across the discovery cohort. A “joint type” is the pair of corresponding joints on opposite sides of the body, e.g., the knee joint type consists of the left and right knee joints. To compute skew for a reference joint type *x* and a co-involved joint type *y*, we counted the number of patients for whom a reference joint was paired with a co-involved joint on the same side (*n*_same_) and on the opposite side (*n*_opposite_) and used these to compute a z-score to measure skew for the joint type pair by setting $z=\frac{m-0.5}{\sigma }$, such that $m=\frac{n_{\mathrm{same}}+1}{n_{\mathrm{same}}+1+n_{\mathrm{opposite}}+1}$ and $\sigma =\sqrt{\frac{m(1-m)}{n}}$. If the joint type pair co-involvement was symmetric, *z* would be close to zero, whereas if co-involved joints tended to occur on the same side of the body, *z* would be large and positive. To assess the significance of the skew, χ^2^ tests were conducted using *n*_same_ and *n*_opposite_ under the null hypothesis that both quantities were equal. *P* values were translated to false discovery rates (FDRs) to account for multiple hypothesis testing. FDR < 0.1 was considered significant.

### Identifying patterns of joint involvement

We selected an analysis strategy suitable for the positive joint counts that would be able to identify sparse patterns of joint involvement if they were present in the data. Sparse patterns better support interpretation and application of these patterns by clinicians. Also, we sought a mixed membership model so that patients could belong to multiple groups. Finally, our preliminary analysis suggested a hierarchical structure to the joint co-involvement patterns, with some tightly coupled joint groups (e.g., toe joints) that often co-occur along with one or more broader patterns of co-activation of these tight groups.

As such, we adapted a form of multilayer non-negative matrix factorization (NMF) [16] to learn hierarchical, sparse representations (S1C Fig). Multilayer NMF progressively applies NMF to the joint patterns. NMF is a dimensionality reduction method that fits summary indicators or “factors” that group frequently co-involved joints together [17]. Broadly, NMF decomposes joint involvements X into a basis/loading matrix W, describing the contributions of joints to factors, and a coefficient/score matrix H that scores patients on factors. Multiplying these two matrices approximates the input data (X ≈ WH). NMF produces an intuitive parts-based representation in which reconstructing patient data involves only adding groups of joints in factors, similar to adding parts of faces (different eyes or noses) to reconstruct facial representations [17]. Unlike other dimensionality reduction techniques like principal component analysis (PCA), NMF constrains the elements of W and H to be positive. This constraint often causes many elements of the two matrices to be zero, a trend that we supplement by setting small elements to zero (a technique called “sparsifying”), as described in S1 Text. NMF can be used to cluster patients by interpreting the nonzero elements of H for each patient as assignments to clusters represented by the factors in W [18].

Multilayer NMF was conducted as described in S1 Text. Briefly, NMF was applied to the joint involvement data, and W was then sparsified to produce low-level factors that correspond to tight joint groupings. NMF was then applied to the H matrix to identify tight joint groupings that often co-occur; the term “high-level factors” refers to these frequently co-occurring tight joint groupings. Through matrix multiplication, joint involvements can be recovered from the high-level factors. “Key joints” for each high-level factor were those appearing with nonzero contributions. For both the high- and low-level factors, patients were assigned into patient groups (“[x]”) corresponding to their highest-scoring factor [19].

The degree of overlap was assessed between patient groups at both levels of the analysis. Patient factor scores were normalized patient-wise to the highest factor score and then z-score–transformed factor-wise. One-sided z-tests determined which patient groups had higher scores than expected on individual factors. FDRs were calculated from *P* values to account for multiple hypothesis testing. Relationships between patient groups and factors were significant if their FDR was <0.1.

### Relationships between patient groups and ILAR subtypes

Relationships were visualized between patient groups and ILAR subtypes through a circular figure built using Circos 0.63 [20]. To identify enriched relationships between patient groups and ILAR subtypes, a χ^2^ test was conducted. Relationships were enriched if their standardized residual was ≥1.96 (i.e., *P* < 0.05).

### Localization of joint involvements

To describe how closely patients aligned with their associated high-level factors or patient groups, patients were further stratified by the “degree of localization” of their active joints. Patients with “localized” involvement had ≥90% of active joints being key joints in the high-level factor underlying their patient group. For patients with “partially localized” involvement, this range was ≥60% and <90%. All other patients had “extended” involvement. S2 Text describes how these boundaries were determined.

To determine which patient groups skewed towards any localization, χ^2^ tests were conducted to compare the distribution of localizations within a single patient group against the global distribution. *P* values were Bonferroni-adjusted for multiple hypothesis testing.

### Associations of patient groups with treatment decisions

For each medication at six-month and one-year visits, multivariable logistic regression was conducted to predict medication status as an outcome from both patient groups and degrees of localization. Model significance was assessed using likelihood-ratio tests. A model for a medication and visit was significant if *P* < 0.05 after a Bonferroni adjustment for multiple hypothesis testing.

### Patient group trajectories and disease trajectories

To track how joint involvement changed over subsequent visits, high-level patient factor scores and patient group assignments were calculated where possible at six-month, one-year, 18-month, two-year, three-year, four-year, and five-year visits. From baseline patient group assignments and localizations, frequencies of transitioning between two patient groups at any time between six-month and five-year visits were calculated. Significantly overrepresented transitions were determined by a 2,000× permutation test.

A multivariate Cox proportional hazards analysis, as implemented in the *survival* R package, version 2.41 [21], was conducted to identify which patient groups, ILAR subtypes, and degrees of localization experienced zero joint involvement more quickly. The assumption of proportional hazards was assessed visually and tested using the “cox.zph” function in *survival*. Hazard ratios (HRs) and 95% confidence intervals (CIs) were computed and patient groups, ILAR subtypes, and degrees of localization were compared using log-rank tests.

### Validation

To determine whether the identified patterns of joint involvement could generalize beyond the discovery cohort, validation cohort joint involvement data were projected onto high-level factors and patient groups. At each level, the same scaling parameters were applied to joint involvement data or low-level patient factor scores. Patients were assigned to patient groups based on their highest-scoring high-level factors as above. Patterns of joint involvement were also identified independently of discovery joint patterns using the multilayer NMF framework described above.

## Results

### Patient characteristics

We included 640 patients with newly diagnosed JIA in the discovery cohort and 119 in the validation cohort. Table 1 outlines demographic data for these cohorts respectively. Discovery cohort patients were diagnosed at a median age of 7.7 years, with a range of 0.57 to 16.6 years, whereas validation cohort patients were diagnosed at a younger age (median: 5.7 years; range: 0.5–18 years). The most highly represented subtype in both cohorts was oligoarthritis (discovery: 47%; validation: 56%), and most patients were female (discovery: 65%; validation: 71%).

**Table 1:** Patient demographics.

Feature	Cohort	
	Discovery	Validation
**Number of patients**	640	119
**ILAR classification, *n* (%)**		
• Systemic arthritis	21 (3.3%)	10 (8.4%)
• Oligoarthritis	300 (47%)	67 (56%)
• RF-negative polyarthritis	125 (20%)	17 (14%)
• RF-positive polyarthritis	16 (2.5%)	5 (4.2%)
• Psoriatic arthritis	37 (5.8%)	6 (5.0%)
• ERA	78 (12%)	13 (11%)
• Undifferentiated arthritis	63 (9.8%)	1 (0.8%)
**Demographic features**		
• Age at diagnosis, years^†^	0.57 ≤ 7.7 ≤ 16.6	0.5 ≤ 5.7 ≤ 18
• Symptom onset to diagnosis, months^†^	0.0 ≤ 3.6 ≤ 12	0 ≤ 0.14 ≤ 1.5
• Female, %	415 (65%)	84 (71%)
**Clinical features**		
• Number of active joints^†^	1 ≤ 2 ≤ 63	1 ≤ 2 ≤ 43
• CHAQ score^†^	0.00 ≤ 0.38 ≤ 3.0	—
• JAQQ score^†^	1.0 ≤ 2.6 ≤ 6.9	—
• QoML score^†^	0.0 ≤ 7.9 ≤ 10	—
• Physician global assessment of disease activity^†^	0.0 ≤ 2.9 ≤ 10	—
**Laboratory parameters**		
• RF-positive, *n*/patients tested (%)	30/551 (5.4%)	6/107 (5.6%)
• ANA-positive, *n*/patients tested (%)	297/579 (51%)	62/112 (55%)
• HLA-B27–positive, *n*/patients tested (%)	58/269 (22%)	9/97 (9.3%)
• ESR, mm/h^†^	0.0 ≤ 19.5 ≤ 130	3.0 ≤ 30 ≤ 102
• CRP, mg/dL^†^	0.0 ≤ 1.7 ≤ 341	0.0 ≤ 3.5 ≤ 409
• Hemoglobin, g/L^†^	8.2 ≤ 121 ≤ 157	—
• Platelets, 10^9^/L^†^	5.9 ≤ 362 ≤ 1,110	—

^†^Minimum ≤ median ≤ maximum.

All patients experienced symptom onset before the age of 16 years.

**Abbreviations:** “—,” not available; ANA, antinuclear antibody; CHAQ, Childhood Health Assessment Questionnaire; CRP, C-reactive protein; ERA, enthesitis-related arthritis; ESR, erythrocyte sedimentation rate; HLA-B27, human leukocyte antigen B27; ILAR, International League of Associations for Rheumatology; JAQQ, Juvenile Arthritis Quality of Life Questionnaire; QoML, Quality of My Life Considering My Health; RF, rheumatoid factor.

### Joints were co-involved in distinct patterns across the entire discovery cohort

**Fig 1:**
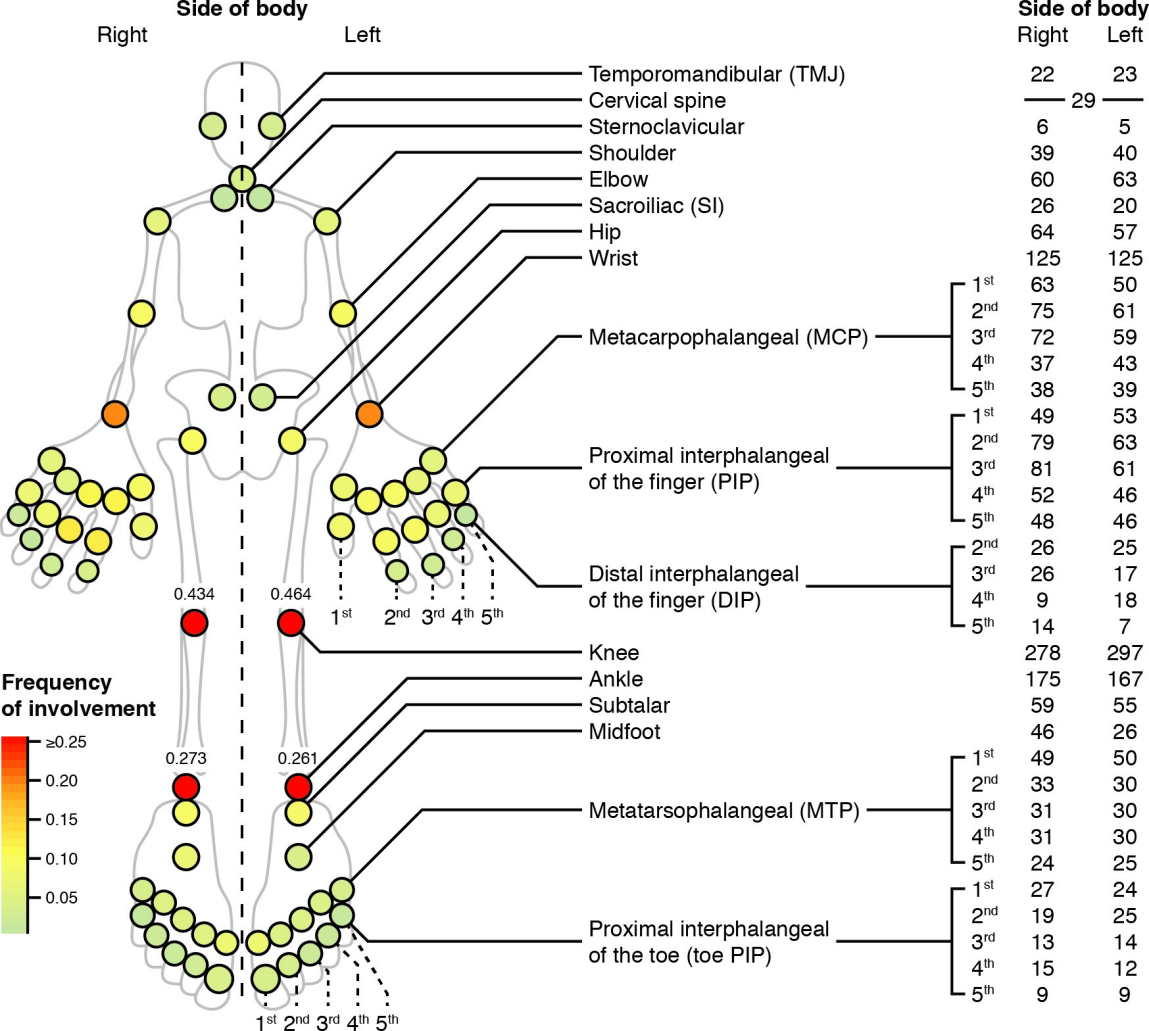
Site involvement frequencies for the discovery cohort. Homunculus of joints (circles) measured in this study, colored by the frequency of involvement (bottom-left legend). Patient numbers are on the right.

**Fig 2:**
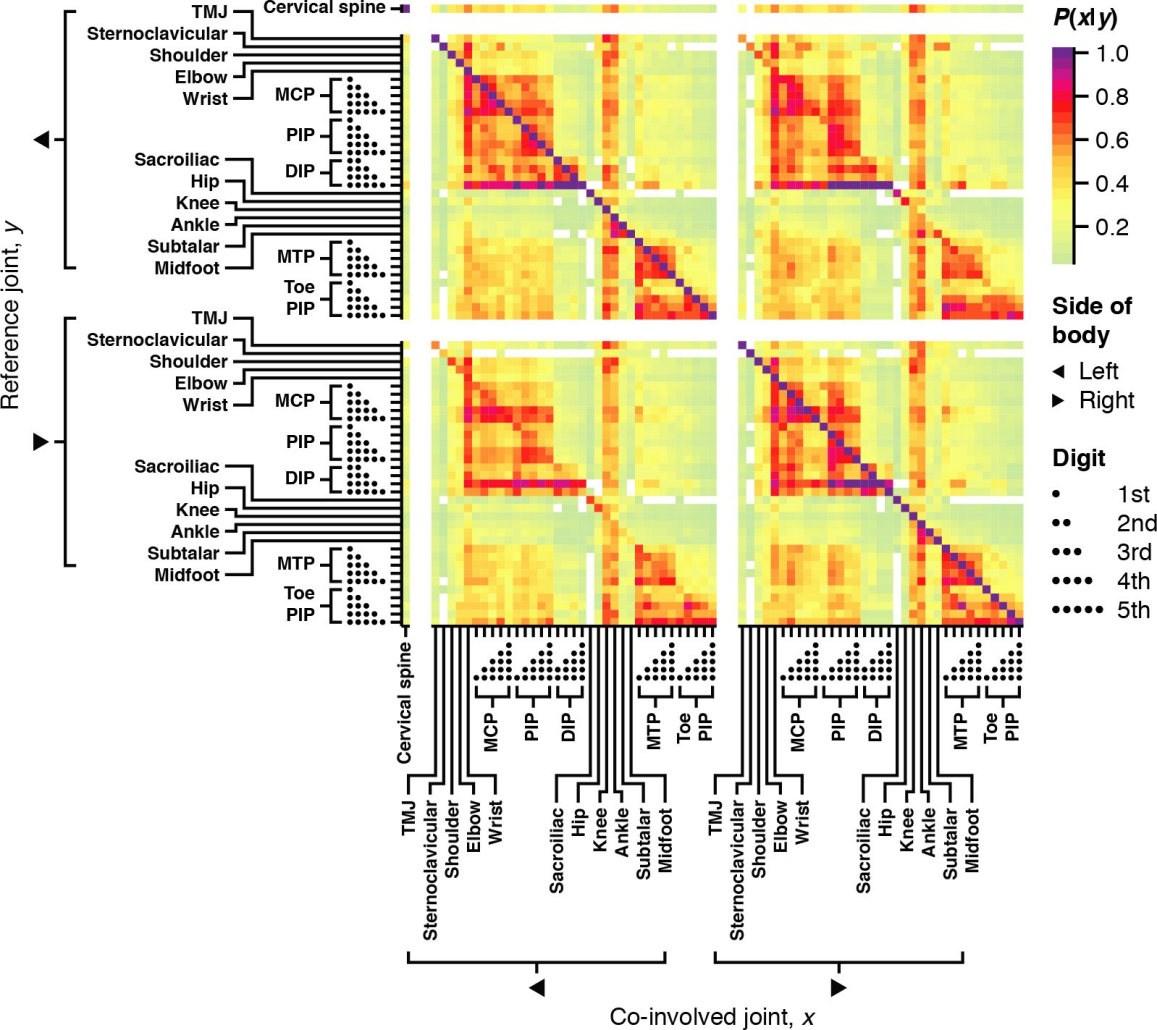
Logical groupings of joints were co-involved. Heat map of conditional probabilities of involvement, *P*(*x*|*y*) (colors; right upper legend), of co-involved joints *x* (x-axis) given reference joints *y* (y-axis). White denotes zero. Arrows represent side of body (right middle legend), and the number of dots represents finger or toe number (right lower legend). DIP, distal interphalangeal; MCP, metacarpophalangeal; PIP, proximal interphalangeal; TMJ, temporomandibular joint.

To investigate patterns of joint involvement and co-involvement, we computed overall frequencies of individual joint involvement and pair co-involvement in the discovery cohort. Fig 1 depicts overall joint involvement frequencies. Knees, ankles, and wrists had the highest rate of involvement. When we considered conditional co-involvement frequencies partitioned by side of body (Fig 2), 3,271 of 5,041 (65%) of these probabilities were significant (*P* < 0.05 after Bonferroni adjustment). Examining the heat maps for same-side joints in the top-left and bottom-right quadrants, we observed hierarchical patterns of joint co-involvement along the vertical axis. Joints closer on the vertical body axis were generally more often co-involved joints. For example, index finger joints and middle finger joints were likely to be co-involved. However, there also appeared to be broad, nonlocal patterns of co-involvement, e.g., finger and toe joints were frequently co-involved.

In contrast, the heat maps for opposite-side involvement in the top-right and bottom-left quadrants appeared nearly identical (Frobenius norm = 3.7; *P* < 0.001 by permutation test), indicating little proximity preference along the horizontal axis. For example, left index finger joints were as likely to be co-involved with right middle finger joints as those on the left middle finger. This observation was consistent with widespread symmetric joint involvement in JIA.

Asymmetric joint involvement is associated with more severe forms of JIA [22]. To study the prevalence of asymmetric co-involvements, we determined which joint types were more likely to be co-involved with joints on the same side of the body. Consistent with our initial impression of Fig 2, and different than what we expected based on previous reports in JIA, we found few statistically significant asymmetric joints: only ankle, midfoot, and subtalar joints (with ankle: χ^2^ = 14, *P*_FDR_ = 0.0097; with subtalar joints: χ^2^ = 11, *P*_FDR_ = 0.048) had statistically significant same-side co-involvement (S2 Fig).

### Multilayer NMF identified seven distinct patterns of joint involvement

To characterize hierarchical patterns of joint co-involvements, we applied multilayer NMF on discovery cohort joint involvements to identify groups of frequently co-involved joints. Conventional NMF identified 19 low-level groupings (or factors)—<1–19> (S6A Fig)—of vertically proximal joints, which S3 Text details. Consistent with the pairwise analysis (S2 Fig), most factors grouped joints of the same type (S6A Fig), with exceptions being pairs of groups containing only ankles and subtalar joints, only a single knee, and one group containing joints from the index (second) and middle (third) fingers on the right side. We named these 19 factors (“<x>”) as follows: <1 TMJs>, <2 shoulders>, <3 sternoclavicular joints>, <4 elbows>, <5 thumbs>, <6 sacroiliac joints>, <7 MCPs>, <8 second–third fingers>, <9 hips>, <10 subtalar and midfoot>, <11 finger PIPs>, <12 knee>, <13 knee>, <14 finger DIPs>, <15 subtalar and midfoot>, <16 grand toes>, <17 ankles>, <18 toe IPs>, and <19 MTPs>. Multiple low-level joint groups were significantly co-involved (S7A Fig), suggesting a hierarchical structure not captured by low-level factors.

To identify this hierarchical structure to the co-involvements, we conducted a second round of conventional NMF on low-level patient scores, which identified seven groupings of groups we deem “high-level factors”, <A–G>. S3 Text describes the process of deriving these high-level factors. As expected from our preliminary investigations (Fig 2), each high-level factor combined low-level factors into broader, symmetric groupings covering partially overlapping areas of the vertical body axis (S6 Fig). We named these high-level factors <A pelvic girdle>, <B fingers>, <C wrists>, <D toes>, <E ankles>, <F knees>, and <G sternoclavicular joints>. Finger distal interphalangeal (DIP) joints distinguished <B fingers> from <C wrists>, as <B fingers> skewed towards finger DIPs and <C wrists> towards shoulders and elbows. <A pelvic girdle> included sacroiliac joints and/or hips, and <E ankles**>** included ankles, subtalar, and midfoot joints.

**Fig 3:**
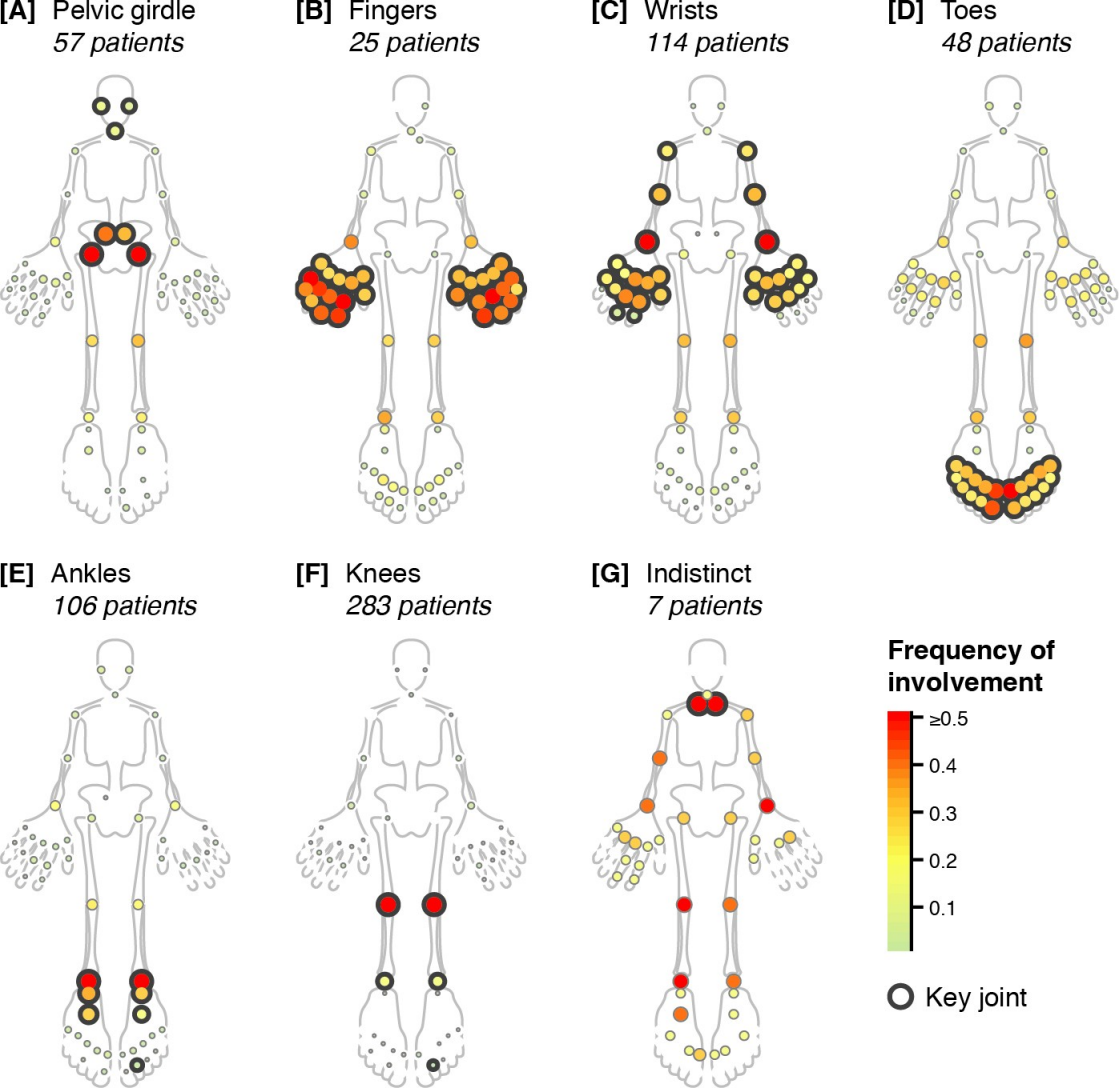
Distinct patterns of joint involvement. Homunculi of frequencies of involvement (colors; bottom-right legend) for individual joints (circles) for each patient group (panels) identified in the discovery cohort. White denotes a frequency of zero. Outlined joints represent key joints for high-level factors (S6 Fig) underlying each patient group. See Fig 1 for the identity of each joint.

We classified each patient into one of seven groups (“[x]”) corresponding to the high-level factors based on their highest-scoring high-level factor. Fig 3 shows joint involvement frequencies in each patient group, and S8 Fig depicts individual joint involvements for patients. Key joints—outlined in Fig 3—were those with nonzero weights in the corresponding high-level factors (S6 Fig). Non-key joints were rarely involved except in [G indistinct] and for some finger/wrist involvement in [D toes], suggesting that most patients had what we deem localized involvement; in other words, most or all of their joints overlapped with key joints. Overall, these patient groups corresponded to logical patterns of joint involvement reported at the bedside [23].

### Patterns of joint involvement subdivided the ILAR subtypes

**Fig 4:**
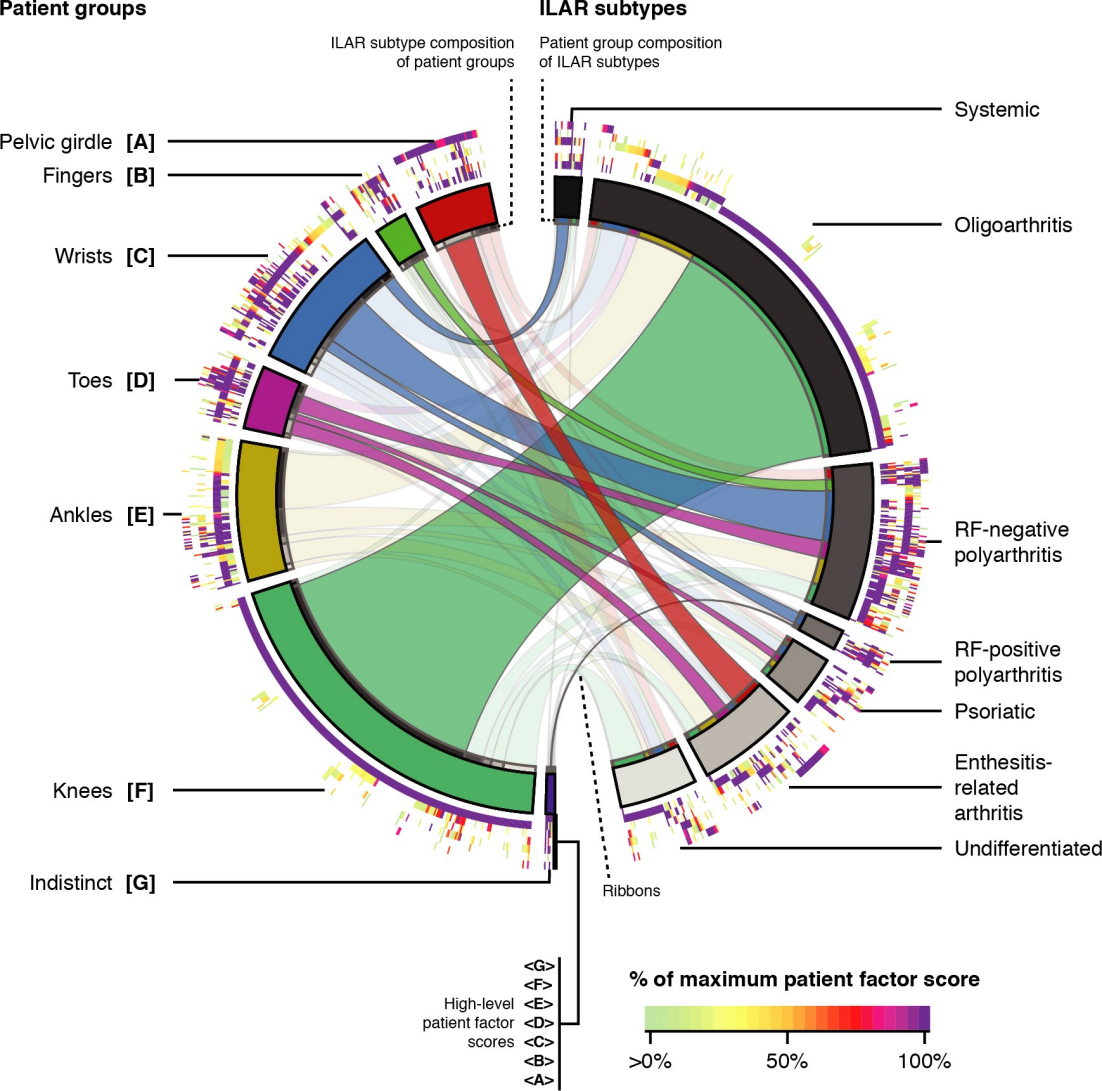
Patient groups subdivided ILAR subtypes. Circos figure visualizing relationships between patient groups (left; colored wedges in the second-innermost ring) and ILAR subtypes (right; grey wedges in the second-innermost ring). Heat maps (outer rings; bottom legend) display high-level patient factor scores, scaled to 0% to 100%, starting with <A pelvic girdle> moving outwards to factor <G sternoclavicular joints>. Each set of arcs along the radial axis, aligned along a ray from the center, represents an individual discovery cohort patient. Ribbons link groups of patients between patient groups and ILAR subtypes. Enriched relationships, as determined from a χ^2^ test, appear more opaque. ILAR, International League of Associations for Rheumatology; RF, rheumatoid factor.

Six of seven patient groups associated with at least one ILAR subtype, despite patient groups comprising different stratifications of patients from the ILAR subtypes (χ^2^ = 313; *P* < 0.001). Conversely, six of seven ILAR subtypes associated with patient groups. More opaque ribbons in the Circos figure (Fig 4), linking patients common to patient groups and ILAR subtypes, represent these enriched associations encompassing more patients than expected through standardized residuals from the above χ^2^ test (S2 Table). These standardized residuals quantify how far the number of patients shared by a patient group and ILAR subtype deviated from expectation. Children in [A pelvic girdle] associated with enthesitis-related arthritis (ERA), [B fingers] with rheumatoid factor (RF)-negative polyarthritis; [C wrists] with systemic arthritis and both RF-positive and negative polyarthritis; [D toes] with RF-negative polyarthritis, psoriatic arthritis, and ERA; and [F knees] with oligoarthritis.

### Some patients had nonlocalized joint involvement

Although the high-level groupings completely encapsulated joint involvements for most patients (56%), a small group of patients (25%) had more non-key joints involved than key joints (S9 Fig). We deemed these patients as having extended involvement. Patients with 90% of their joints as key were localized, and those with 60% to 90% were partially localized. S4 Text describes how we determined these thresholds. To determine the clinical significance of these subcategories, we compared the clinical attributes of patients therein.

Patient groups with significantly skewed distributions of localizations included [C wrists], [D toes], [F knees], and [G indistinct] (χ^2^ ≥ 21.5; *P* < 0.001; S9C Fig and S3 Table). Patients in [G indistinct] skewed towards extended involvement, [C wrists] towards partially localized involvement, and [D toes] towards both partially localized and extended involvement. Children in [F knees] skewed towards localized involvement.

### Treatment decisions were associated with degrees of localization

To determine whether treatment decisions were associated with patient groups and degrees of localization, we conducted multivariable logistic regression. S10A Fig depicts the number of patients in each group and localization with medication data at six-month and one-year visits. S10B Fig depicts the proportion of patients in each patient group, divided by localization, who received biologics, disease-modifying antirheumatic drugs (DMARDs), joint injections, and systemic corticosteroids prior to these visits. Patient groups differed by DMARD usage, with higher than expected utilization in children in [C wrists] and [E ankles] with localized involvement and [F knees] with partially localized and extended involvement, and lower than expected utilization in [F knees] with localized involvement (S4 Table). In terms of systemic corticosteroid usage, patients in [F knees] with localized involvements were less likely to receive such treatment at six-month visits. Therefore, patient joint group and localization influenced treatment.

### Patient groups remained stable throughout initial disease course

**Fig 5:**
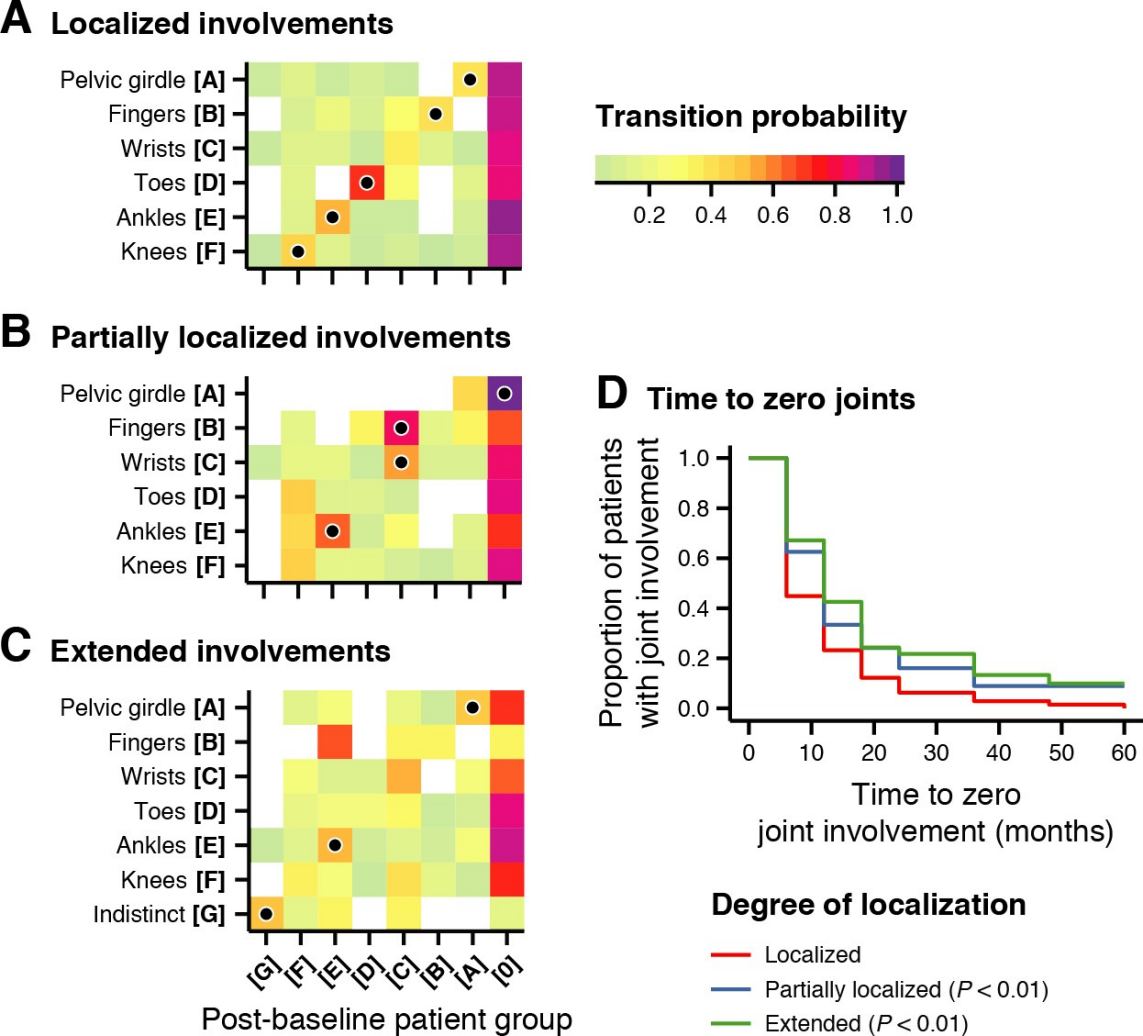
Patient groups and localizations predicted disease course. (A) Heat maps showing the probability (colors; bottom legend) that patients in a baseline or reference patient group (y-axis) with localized involvement transition to patient groups at any visit from six months to five years (x-axis). Filled circles denote significantly enriched transitions (*P* < 0.05; Holm-Bonferroni–adjusted). (B) Same as panel A, but for patients with partially localized involvement. (C) Same as panel A, but for patients with extended involvement. (D) Time to zero curves, by degree of localization (line types; bottom legend), showing the proportion of patients continuing to have joint involvement (y-axes) after given visits after baseline (x-axes). *P* values represent those for HRs calculated by a Cox proportional hazards model. HR, hazard ratio.

To observe how the data-driven joint patterns evolved throughout disease course, we traced patient group assignments longitudinally. Fig 5 depicts transition probabilities from baseline patient groups, divided by degree of localization, to groups at any visit up to five years. While patients often transitioned to zero joint involvement, several transitions were enriched (*P* < 0.05; Holm-Bonferroni–adjusted), with little movement between patient groups given the lack of filled circles outside the diagonals. Among patients with localized involvements (Fig 5A), patients in [A pelvic girdle], [B fingers], [D toes], [E ankles], and [F knees] remained in their own patient group. Even among patients with partially localized involvements (Fig 5B), patients in [A pelvic girdle] often transitioned to zero joint involvement, whereas patients in [B fingers] and [C wrists] transitioned to or remained [C wrists]. Patients in [E ankles] often remained in the same patient group. Among patients with extended involvement (Fig 5C), patients in [A pelvic girdle], [E ankles], and [G indistinct] often remained in their respective patient groups.

### Localizations predicted time to zero joint involvement

Having observed a strong tendency for some groups towards zero joint involvement (disease control/inactive disease), we asked whether the groups differed by the rate in which they achieved this outcome. We constructed a Cox proportional hazards model predicting time to zero joint involvement from patient groups, localizations, and ILAR subtypes. The resulting model did not deviate from the proportional hazards assumption (χ^2^ = 22.9, *P* = 0.062) and identified localizations and ILAR subtypes that reached zero joint involvement at different rates than others (*R*^2^ = 0.093, *P* < 0.001). At least half of the patients followed in [F knees] reached this endpoint by six months, and [A pelvic girdle], [C wrists], [D toes], and [E ankles] reached it by one year (S11A Fig). In contrast, patients in [B fingers] reached this endpoint after 18 months, and those in [G indistinct] reached it after three years. Localization was especially important because patients with localized involvement achieved this outcome faster than patients with partial involvement (HR = 0.70, *Z* = −3.1, *P* = 0.0018) and extended involvement (HR = 0.63, *Z* = −2.8, *P* = 0.0057) (Fig 5D). Among diagnoses, patients with RF-positive polyarthritis (HR = 0.42, *Z* = 2.4, *P* = 0.016) and undifferentiated arthritis (HR = 0.64, *Z* = −2.8, *P* = 0.0059) reached this outcome more slowly. S5 Table contains additional statistics for the Cox proportional hazards model.

### Patient groups generalized to an independent validation cohort

To determine whether factors and patient groups were generalizable beyond the discovery cohort, we projected an independent validation cohort of JIA patients to the joint patterns. These patients’ joint involvements were reconstructed by low-level factors with *Q*^2^ = 0.81, high-level factors with *Q*^2^ = 0.55, and patient groups with *Q*^2^ = 0.48 (S1 Table), comparing favorably against ILAR subtypes, with *Q*^2^ = 0.43. Projected patient groups presented with similar joint involvement frequencies (S12 Fig). We then calculated de novo factors and patient groups, which we detail in S4 Text. Validation joint involvements were reconstructed by low-level factors with *Q*^2^ = 0.84, high-level factors with *Q*^2^ = 0.55, and patient groups with *Q*^2^ = 0.35. De novo factors described similar joint groupings as discovery factors (S13C, S13F and S13G Fig).

We then asked whether the projected groups could also predict time to zero joint involvement. Considering individual univariate models due to limited power, patient groups (χ^2^ = 17, *P* = 0.0072) and localizations (χ^2^ = 8.9, *P* = 0.012; S14 Fig) themselves predicted this outcome. Patients with extended involvement took significantly longer to reach zero joint involvement than patients with localized involvement (HR = 0.53, *Z* = −2.5, *P* = 0.011).

## Discussion

We explored patterns of articular involvement in JIA using unsupervised data-driven pattern recognition techniques. We initially observed joints co-occurring in logical and localized groupings without same-side skewing (Figs 1 and 2 and S2 Fig). To better understand these signals in a clinically applicable manner, we conducted a modified version of multilayer NMF, identifying seven high-level groupings of joints (S6 Fig and Fig 3) describing arthritis foci anchored by distinct subsets of joints. The resulting seven patient groups subdivided the ILAR subtypes into distinct subgroups based on patterns of arthritis (Fig 4). Patients with localized involvement often remained in the same patient group after baseline visit (Fig 5A, 5B and 5C) and reached zero joint involvement faster than patients with nonlocalized involvement (Fig 5D). These patterns generalized to an independent validation cohort, supporting their applicability beyond the discovery cohort (S12 Fig and S13 Fig).

Our study is the first to provide a detailed, data-driven description of heterogeneously co-involved joints in JIA. Previous studies have focused on individual joints [2–8] in specific individual ILAR subtypes [9,10], whereas we identified joint groupings in a data-driven fashion independently of these ILAR subtypes. For example, our approach identified differences in presentation among patients with polyarticular involvement, separating these patients based on joint patterns. For example, the small joints of the fingers in [B fingers] and [C wrists] were clearly distinct from the small joints of the toes in [D toes]. The composition of patient groups in RF-negative polyarthritis further supported this distinction, with this ILAR subtype associating with [B fingers], [C wrists], and [D toes]. [B fingers] and [C wrists] may represent a spectrum of disease phenotypes (S7 Fig) that may transition between each other during disease evolution (Fig 5). Wrist involvement has been associated with poor prognosis and decisions to treat more aggressively [6]. Our results reflected this trend as patients in [C wrists] had less defined disease trajectories and longer times to zero joint involvement (S11 Fig) despite more commonly receiving DMARDs and systemic glucocorticoids. Similar findings were observed in patients in [B fingers] which tended to transition into [C wrists], with higher use of (biologic) DMARDs and systemic glucocorticoids at diagnosis. These findings were notable as patients in [B fingers] tended to transition to [C wrists] when they had partially localized involvement.

The stability of the patient groups for up to five years after diagnosis supports how meaningful these patterns are. As patients lose active joints over the course of treatment, we expected their joint patterns to shift as patient factor scores represent weighted sums of individual joints. With fewer active joints, we expected patterns to become more sensitive to the specific joints involved. However, patients remaining in their same groups in at least one subsequent visit suggested that patients with residual joint involvement had it in their group’s key joints. Therefore, joint patterns may represent robust core groupings of joints much like the indicator joints of poor outcome [2–7].

Our study is also among the first to identify the degree of active joint localization with outcomes through an easily measurable clinical variable, the degree of localization. Patients had worse disease outcomes if their active joints did not align strictly with a single pattern. This has been instinctively recognized at the bedside by clinicians, as this aspect has clearly influenced treatment decisions (S4 Table and S10 Fig), but is not included in any clinical guidelines. Furthermore, classifying patients by the degree of localization predicted disease course and time to zero joint involvement. For patients with recognizable patterns of joint involvement, treatment decisions appear effective. However, patients with partially localized or extended joint involvement had the poorest outcomes, taking a longer time to reach zero joint involvement despite receiving stronger medications (e.g., more DMARDs). Patients with nonlocalized joint involvement may therefore represent a high-risk group who require early aggressive therapy.

Our pattern recognition approach is well suited for analyzing joint involvements. Factors supported signals that were apparent based on overall co-involvements. Low-level factors grouped knees, subtalar joints, and midfoot joints on separate sides of the body into separate factors, demonstrating that our approach identifies both asymmetrical and symmetrical patterns of arthritis. As our approach identified patterns with little overlap outside the fingers, patient groups described clinically homogeneous entities. Extending NMF into a multilayer approach bears some similarity to other hierarchical modelling techniques such as deep autoencoders [24], in which each successive layer identifies increasingly broad representations of the data, or Gaussian process latent variable models [25]. However, multilayer NMF provides directly interpretable latent representations. This representation differs from PCA, which produces patterns that are orthogonal to each other, a feature with implications with respect to joint involvements. Furthermore, PCA patterns are less intuitive as joints contribute both positively and negatively to them. To reconstruct a patient’s joint involvements, we would have to add and subtract groups of joints, whereas with NMF, we would only add groups of joints.

Our study has a number of limitations. First, both discovery and validation cohorts were based on sample sizes of convenience. Because discovering joint patterns involved an unsupervised analysis, a priori power analyses were not done. However, the proximity of joints within patterns along the vertical axis and our ability to identify useful clinical measures demonstrated the potential of conducting such an analysis retrospectively. Secondly, the small size of the validation cohort limited our ability to test our findings in a multivariate model in a validation cohort, although we successfully validated our predictors in univariate tests. Furthermore, our validation cohort strongly supported these clinical measures, demonstrating that this concern is one of statistical power rather than approach. Lastly, we required patients to be treatment naïve except NSAIDs, which may have potentially skewed our patient cohorts towards individuals with milder forms of disease.

The identified joint patterns appear to have important prognostic implications. They are conceptually simple to apply at the bedside as they represent an easily computed weighted sum of active joints. Further classifying patients by the degree of localization may help clinicians further tailor treatment decisions as patients with less strongly defined phenotypes may require early aggressive therapy. Patterns of joint involvement may be among key components of a new disease classification for JIA in addition to other data domains, including antinuclear antibody (ANA) status [26], biological measures [13], and other musculoskeletal features such as enthesitis. Beyond JIA, our approach may be a transferrable template for application in rheumatoid arthritis (RA) and spondyloarthropathies (SpAs). Previous efforts in RA have attempted to define a representative pattern of “core joints” as indicators instead of using the full complement of joints [27,28]. This reductionist approach still counts joints. Alternatively, utilizing the rich data for pattern recognition may identify predictors of outcome.

Using multilayer NMF, we identified patterns of joint involvement predictive of disease trajectory in children with arthritis. Our results are consistent with previous observations pointing to key individual joints as predictors of poor outcome. Our hierarchical unsupervised learning approach allowed us to identify a new clinical variable, localization of joint involvement, as a key feature associated with poor outcomes in both our discovery and validation cohorts. Detailed bedside assessment of every joint is already part of every musculoskeletal exam for children with arthritis. Our study supports not only the continued collection of detailed information about joint involvement but also the inclusion of these patterns together with localization data (i.e., how closely affected children fit these patterns) to stratify patients and inform treatment decisions. Our findings will move the field of pediatric rheumatology out of infancy, from joint counts to realizing the potential of using data available from patterns of joints involvement.

## Supporting information

S1 Checklist TRIPOD checklist. TRIPOD, transparent reporting of a multivariable prediction model for individual prognosis or diagnosis.
(DOCX)

S1 Fig Analysis workflow. (A) Overall analysis workflow for the discovery and validation cohorts to identify factors and patient groups from joint involvement data. (B) Factor identification workflow, which considers input data (left) comprising joint involvements or low-level factor scores and identifies factors described by coefficient/score matrices and basis/loading matrices (right). (C) The overall multilayer NMF scheme for this study. Boxes represent layers vertically scaled to the number of dimensions, which are, from left to right, the number of joints, the number of low-level factors, and the number of high-level factors. Circles represent patient groups. NMF, non-negative matrix factorization.
(TIF)

S2 Fig Few pairings of joint types were asymmetrically involved. Heat maps of z-scores (colors; right legend), for co-occurring joint types (x-axis) on reference joints (y-axis). Pairings whose absolute z-score was <1 were zeroed. Pairings with FDR < 0.1 are outlined. FDR, false discovery rate.
(TIF)

S3 Fig Nineteen low-level and seven high-level factors in the discovery cohort. (A) Reconstruction accuracy of joint involvement data (first-level analysis) based on number of low-level factors (x-axis) using 2,000× 3-fold BiCV. *Q*^2^ (y-axis) correlates, throughout BiCV, withheld data with their original values. Higher *Q*^2^ is better. (B) Reconstruction accuracy of joint involvement data (first-level analysis) based on regularization coefficient (*α*; x-axis) using 2,000× 3-fold BiCV. The number of factors was fixed to 19. The horizontal grey strip represents the standard error when *α* = 0. (C) Same as panel A, but for the number of high-level factors (x-axis) with respect to low-level patient factor scores. (D) Same as panel B, but for high-level factors with respect to low-level patient factor scores. The number of factors was fixed to seven. BiCV, bi-cross-validation.
(TIF)

S4 Fig Sparsification maintained relationships between patients on factors. (A) Scatterplots of sparsified scores (y-axes) compared to unsparsified scores (x-axes) for each low-level factor (subpanel). Each point represents a single patient colored by the density of patients within its vicinity (bottom legend). Diagonal lines represent lines of best fit. For all low-level factors, *P* < 0.001. *ρ*: Spearman correlation. (B) Same as panel A, but for high-level factors (subpanels). For all high-level factors, *P* < 0.001.
(TIF)

S5 Fig Factors described localized groupings of joints. (A) Heat map of unsparsified contributions of individual joints (y-axis) to low-level factors (x-axis). Contributions of sites to factors, scaled to 0%–100%, are given by colors (bottom legend). White denotes 0%. Left and right arrows (bottom-right legend) denote side of body. (B) Same as panel A, but for high-level factors (x-axis). (C) Same as panel B, but for unsparsified contributions of low-level factors (x-axis) to high-level factors (y-axis).
(TIF)

S6 Fig Sparsified factors described localized groupings of joints. (A) Heat map of sparsified contributions of individual joints (y-axis) to low-level factors (x-axis). Contributions of sites to factors, scaled to 0%–100%, are given by colors (bottom legend). White denotes 0%. Left and right arrows (bottom-right legend) denote side of body. (B) Same as panel A, but for high-level factors (x-axis). (C) Same as panel B, but for sparsified contributions of low-level factors (x-axis) to high-level factors (y-axis).
(TIF)

S7 Fig Factors and patient groups displayed little overlap. (A) Heat map of negative log-FDRs (colors; bottom-right legend) depicting the degree to which low-level patient groups (x-axis) associated with low-level factors (y-axis) based on z-tests. White denotes a negative log-FDR less than 1.3 (i.e., FDR < 0.05). (B) Same as panel A, but for high-level patient groups (x-axis) and high-level factors (y-axis). FDR, false discovery rate.
(TIF)

S8 Fig Distinct joint patterns with respect to individual joints. Heat map of individual joint involvements (x-axis) for each discovery cohort patient (y-axis), grouped by patient group (rows). Black cells indicate active joints that appear in high-level factors, or key joints, underlying patient groups (S6B Fig). Grey cells indicate other active joints (bottom-left legend). Left and right arrows (bottom-right legend) denote side of body. DIP, distal IP; IP, interphalangeal; MCP, metacarpophalangeal; MTP, metatarsophalangeal; PIP, proximal IP; TMJ, temporomandibular joint.
(TIF)

S9 Fig Thresholds for distinguishing degrees of joint localization. (A) At each given threshold (x-axis), the proportion of patients exceeding that threshold (y-axis). This threshold describes, for a given patient, the proportion of joints that are also key joints of that patient’s underlying high-level factor (S6B Fig). Error bars represent standard errors derived from 2,000 bootstraps. (B) Same as panel A but divided into patient groups (subpanels). (C) Bar graph of the percent of patients in each group with each localization (shades of grey; right legend). Up arrows denote enriched combinations of patient groups and localizations, and down arrows denote depleted or rarer combinations. ***P < 0.001 by χ^2^ test with Bonferroni correction for multiple hypothesis testing.
(TIF)

S10 Fig Treatment decisions associated with degrees of localizations. (A) Bar plots of the number of patients followed (y-axis) per patient group (columns) per visit (shades of grey; bottom legend). (B) Bar plots showing, for each treatment (rows), the proportion of patients (y-axes) per patient group (columns) and degree of localization (shades of grey; bottom legend) prescribed that treatment within six-month windows prior to six-month and one-year visits (x-axes). Up arrows denote, for each patient group, visit, and patient group, enriched localizations that occur more often than expected, and down arrows denote depleted localizations that occur less often than expected.
(TIF)

S11 Fig Patient groups reached zero joint involvement at different rates. Time to zero curves for each patient group (columns) showing the proportion of patients continuing to have joint involvement (y-axes) after given visits after baseline (x-axes) and degree of localization (colors; bottom legend).
(TIF)

S12 Fig Joint patterns generalized to an independent validation cohort. Homunculi of frequencies of involvement (colors; bottom-right legend) for individual joints (circles) for each projected patient group (panels) in the validation cohort. White denotes a probability of zero. See Fig 1 for the identity of each joint.
(TIF)

S13 Fig Similar de novo joint patterns in an independent validation cohort. (A) Mean reconstruction accuracies (*Q*^2^; y-axis) of increasing numbers of low-level factors (x-axis). The highest *Q*^2^ is circled, and the corresponding number of factors is indicated by the dashed vertical line. Error bars represent 95% CIs. (B) Same as panel A, except for regularization coefficient (x-axis). The dashed horizontal line represents the mean *Q*^2^ when the regularization constant is zero, and the grey ribbon represents its standard deviation. (C) Same as panel A, except for high-level factors. (D) Same as panel B, except for high-level factors. (E) Heat map of contributions (colors; right lower legend) of joints (y-axis) to low-level factors (x-axis). White denotes zero contributions. Arrows denote side of body (right upper legend). (F) Heat map of contributions (colors; right lower legend) of low-level factors (x-axis) to high-level factors (y-axis). White denotes zero contributions. (G) Same as panel E, but for contributions of joints (y-axis) to high-level factors (x-axis).
(TIF)

S14 Fig Disease course for the validation cohort. Time to zero curves, by degree of localization (colors; bottom legend), showing the proportion of patients continuing to have joint involvement (y-axes) after given visits after baseline (x-axes).
(TIF)

S1 Table Reconstruction accuracy (*Q*^2^) of joint involvement data by factors, patient groups, and ILAR subtypes. ILAR, International League of Associations for Rheumatology.
(DOCX)

S2 Table Associations between patient groups and ILAR subtypes. Standardized residuals *ϵ* generated from a χ^2^ test on a contingency table counting patients at the intersection of each patient group and ILAR subtype. *P < 0.05, **P < 0.01, ***P < 0.001. ILAR, International League of Associations for Rheumatology; RF, rheumatoid factor.
(DOCX)

S3 Table Associations between patient groups and degrees of localization. *P < 0.05, **P < 0.01, ***P < 0.001.
(DOCX)

S4 Table Associations between patient groups and degrees of localization and treatment decisions prior to subsequent visits. Associations are enriched, or observed more often than expected, when the coefficient *β* > 0, and depleted when *β* < 0. DMARD, disease-modifying antirheumatic drug.
(DOCX)

S5 Table Coefficients from Cox proportional hazards modelling for time to zero joint involvement. *P < 0.05, **P < 0.01. CI, confidence interval.
(DOCX)

S1 Text Multilayer NMF. NMF, non-negative matrix factorization.
(DOCX)

S2 Text Localizations. (DOCX)

S3 Text Logical patterns of joint involvement. (DOCX)

S4 Text Degrees of joint localization. (DOCX)

S5 Text De novo validation. (DOCX)

## Abbreviations

ACR: American College of Rheumatology
ANA: antinuclear antib
CHAQ: Childhood Health Assessment Questionnaire
CI: confidence interval
CRP: C-reactive protein
DIP: distal interphalangeal
DMARD: disease-modifying antirheumatic drug
ERA: enthesitis-related arthritis
ESR: erythrocyte sedimentation rate
FDR: false discovery rate
HLA-B27: human leukocyte antigen B27
HR: hazard ratio
ILAR: International League of Associations for Rheumatology
JAQQ: Juvenile Arthritis Quality of Life Questionnaire
JIA: juvenile idiopathic arthritis
JRA: juvenile rheumatoid arthritis
MCP: metacarpophalangeal
NMF: non-negative matrix factorization
NSAID: nonsteroidal anti-inflammatory drug
PCA: principal component analysis
PIP: proximal interphalangeal
QoML: Quality of My Life Considering My Health
RA: rheumatoid arthritis
ReACCh-Out: Research in Arthritis in Canadian Children, Emphasizing Outcomes
RF: rheumatoid factor
SickKids: The Hospital for Sick Children (Toronto, Ontario, Canada
SpA: spondyloarthropathy
TMJ: temporomandibular joint
TRIPOD: transparent reporting of a multivariable prediction model for individual prognosis or diagnosis
