# Supplementary figures and images for: Patterns of joint involvement in juvenile idiopathic arthritis and prediction of disease course: A prospective study with multilayer non-negative matrix factorization
Source: PLoS Med. 2019 Feb 26;16(2):e1002750. doi: 10.1371/journal.pmed.1002750 (PMC6390994; doi:10.1371/journal.pmed.1002750)

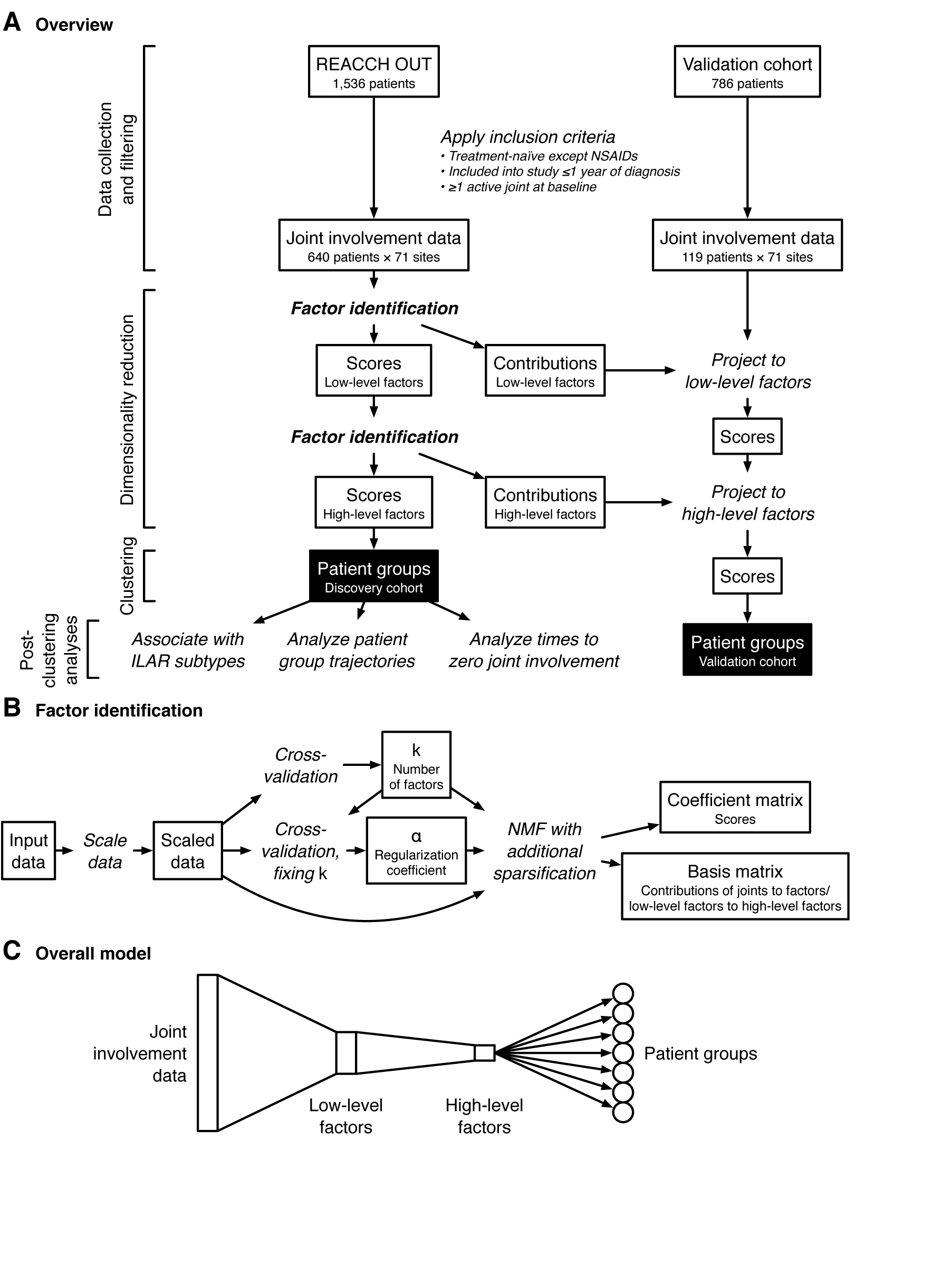

Supplement: S1 Fig — (A) Overall analysis workflow for the discovery and validation cohorts to identify factors and patient groups from joint involvement data. (B) Factor identification workflow, which considers input data (left) comprising joint involvements or low-level factor scores and identifies factors described by coefficient/score matrices and basis/loading matrices (right). (C) The overall multilayer NMF scheme for this study. Boxes represent layers vertically scaled to the number of dimensions, which are, from left to right, the number of joints, the number of low-level factors, and the number of high-level factors. Circles represent patient groups. NMF, non-negative matrix factorization. (TIF) [file pmed.1002750.s002.tif]

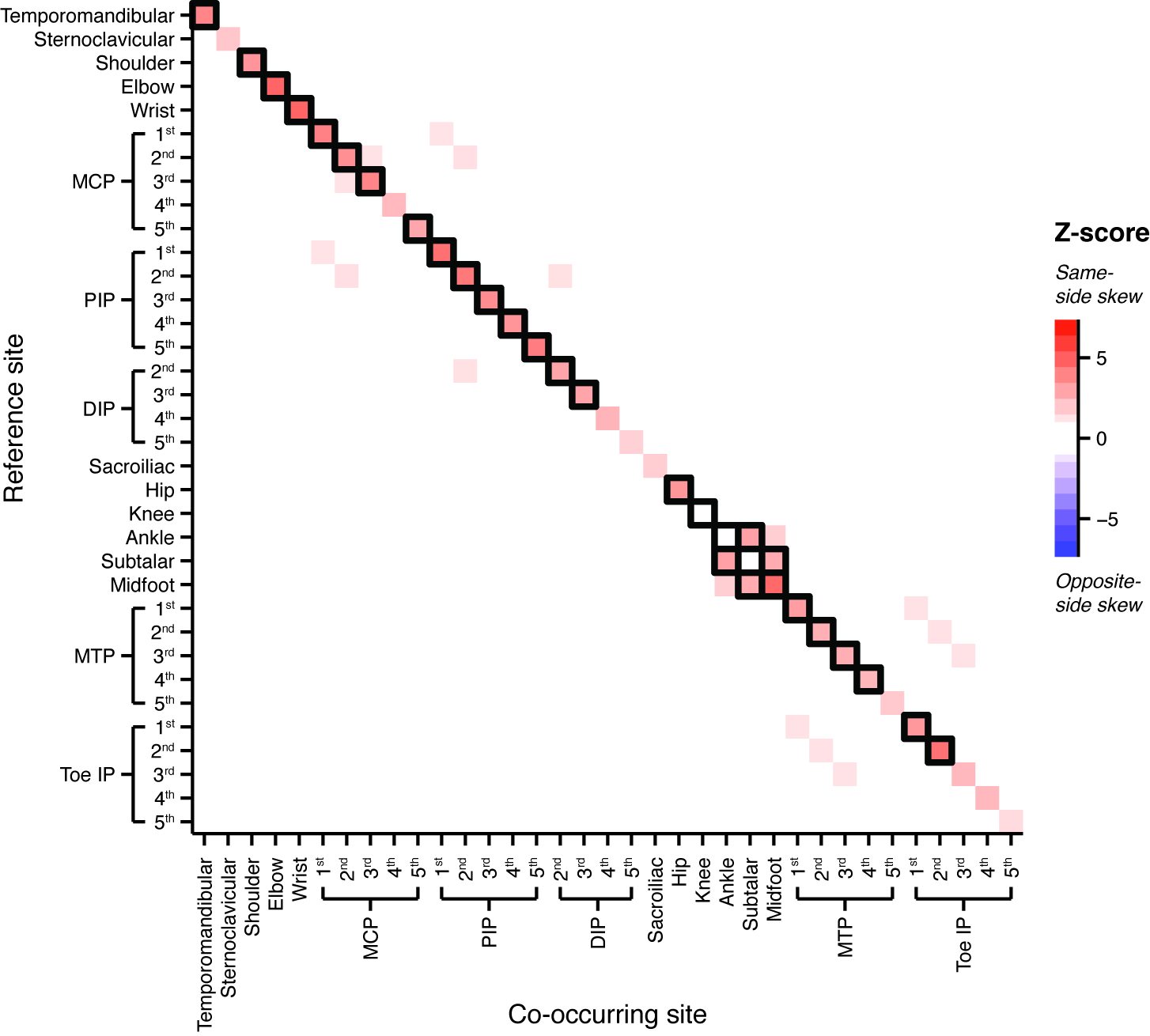

Supplement: S2 Fig — Heat maps of z-scores (colors; right legend), for co-occurring joint types (x-axis) on reference joints (y-axis). Pairings whose absolute z-score was <1 were zeroed. Pairings with FDR < 0.1 are outlined. FDR, false discovery rate. (TIF) [file pmed.1002750.s003.tif]

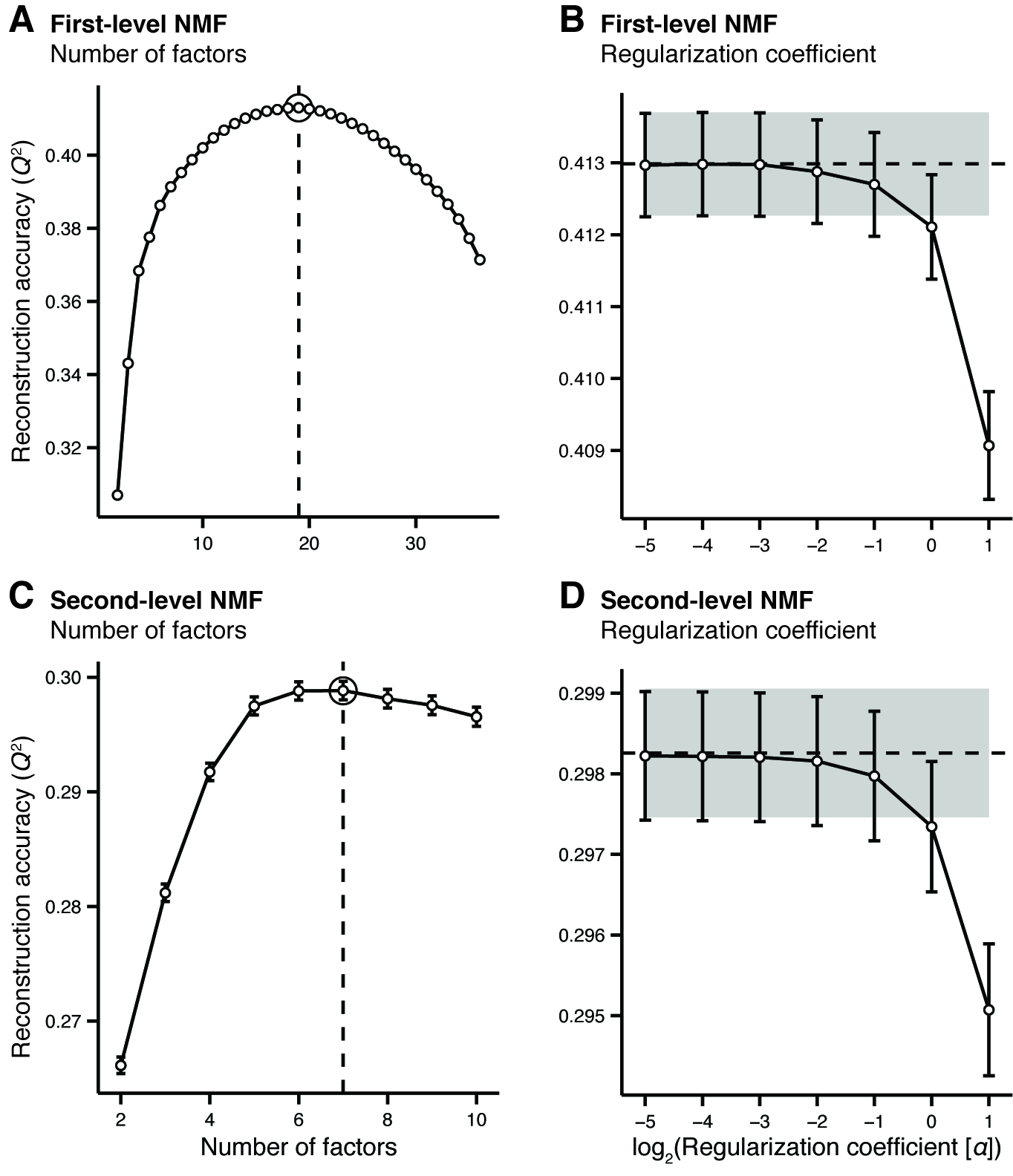

Supplement: S3 Fig — (A) Reconstruction accuracy of joint involvement data (first-level analysis) based on number of low-level factors (x-axis) using 2,000× 3-fold BiCV. Q2 (y-axis) correlates, throughout BiCV, withheld data with their original values. Higher Q2 is better. (B) Reconstruction accuracy of joint involvement data (first-level analysis) based on regularization coefficient (α; x-axis) using 2,000× 3-fold BiCV. The number of factors was fixed to 19. The horizontal grey strip represents the standard error when α = 0. (C) Same as panel A, but for the number of high-level factors (x-axis) with respect to low-level patient factor scores. (D) Same as panel B, but for high-level factors with respect to low-level patient factor scores. The number of factors was fixed to seven. BiCV, bi-cross-validation. (TIF) [file pmed.1002750.s004.tif]

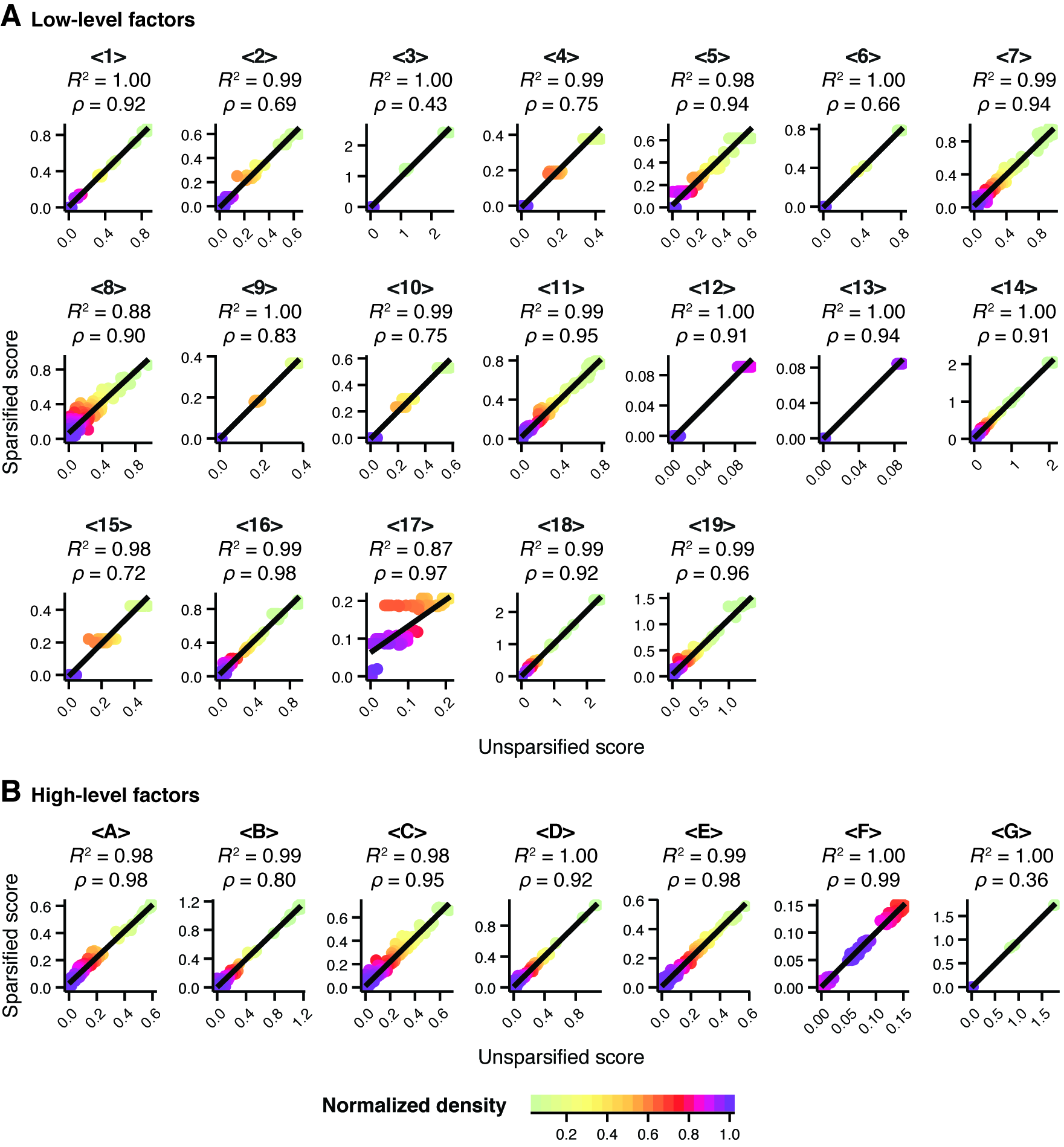

Supplement: S4 Fig — (A) Scatterplots of sparsified scores (y-axes) compared to unsparsified scores (x-axes) for each low-level factor (subpanel). Each point represents a single patient colored by the density of patients within its vicinity (bottom legend). Diagonal lines represent lines of best fit. For all low-level factors, P < 0.001. ρ: Spearman correlation. (B) Same as panel A, but for high-level factors (subpanels). For all high-level factors, P < 0.001. (TIF) [file pmed.1002750.s005.tif]

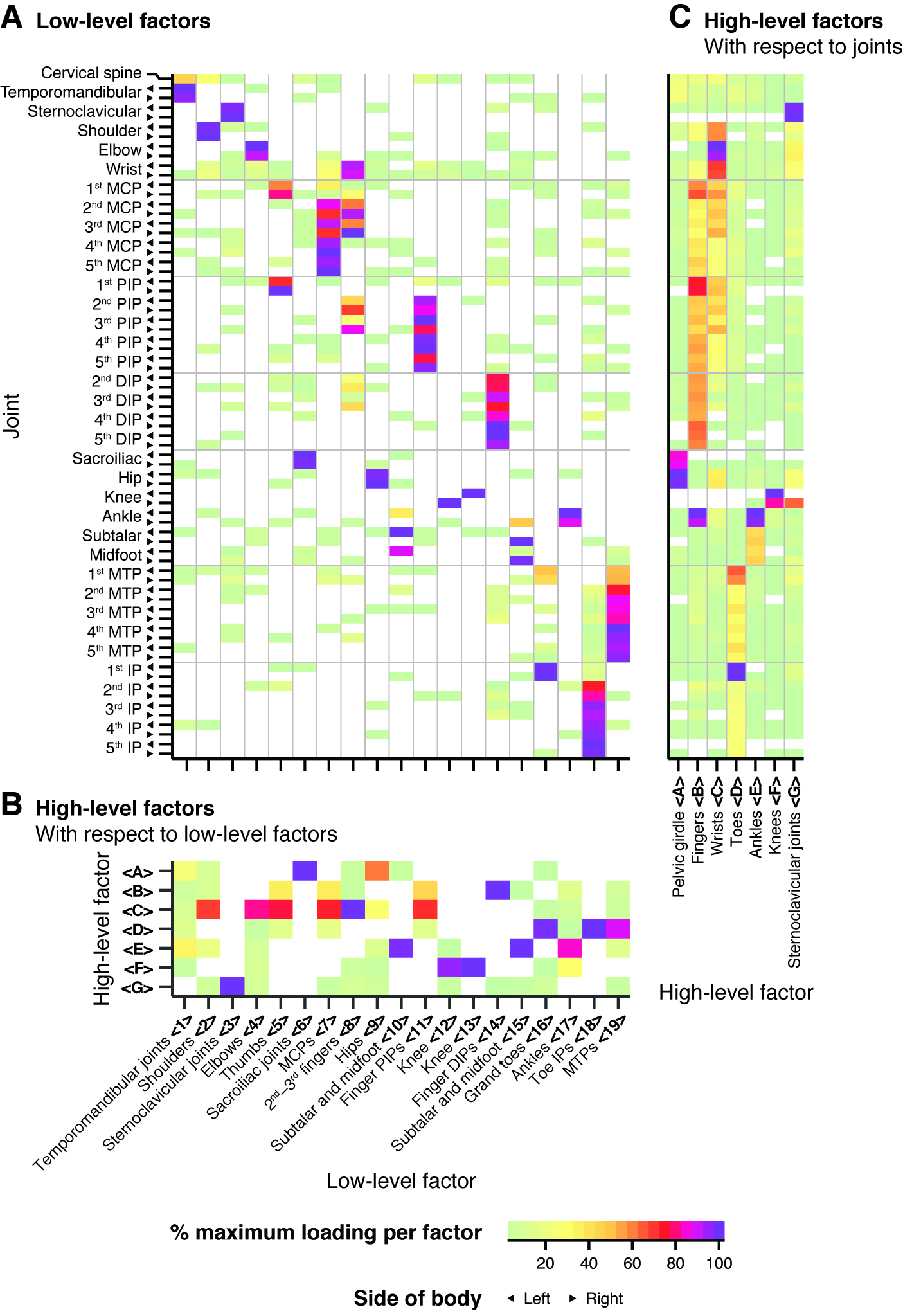

Supplement: S5 Fig — (A) Heat map of unsparsified contributions of individual joints (y-axis) to low-level factors (x-axis). Contributions of sites to factors, scaled to 0%–100%, are given by colors (bottom legend). White denotes 0%. Left and right arrows (bottom-right legend) denote side of body. (B) Same as panel A, but for high-level factors (x-axis). (C) Same as panel B, but for unsparsified contributions of low-level factors (x-axis) to high-level factors (y-axis). (TIF) [file pmed.1002750.s006.tif]

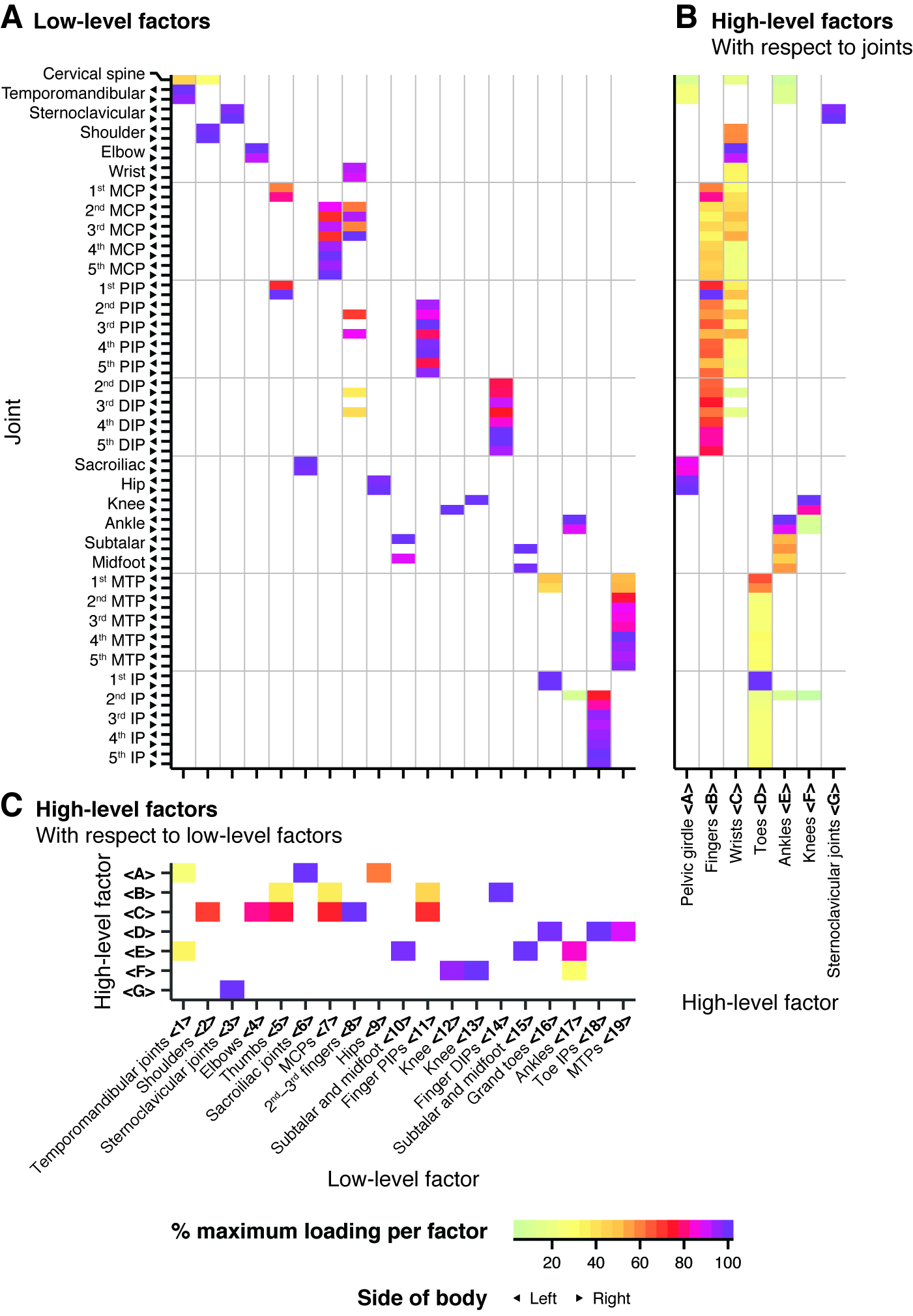

Supplement: S6 Fig — (A) Heat map of sparsified contributions of individual joints (y-axis) to low-level factors (x-axis). Contributions of sites to factors, scaled to 0%–100%, are given by colors (bottom legend). White denotes 0%. Left and right arrows (bottom-right legend) denote side of body. (B) Same as panel A, but for high-level factors (x-axis). (C) Same as panel B, but for sparsified contributions of low-level factors (x-axis) to high-level factors (y-axis). (TIF) [file pmed.1002750.s007.tif]

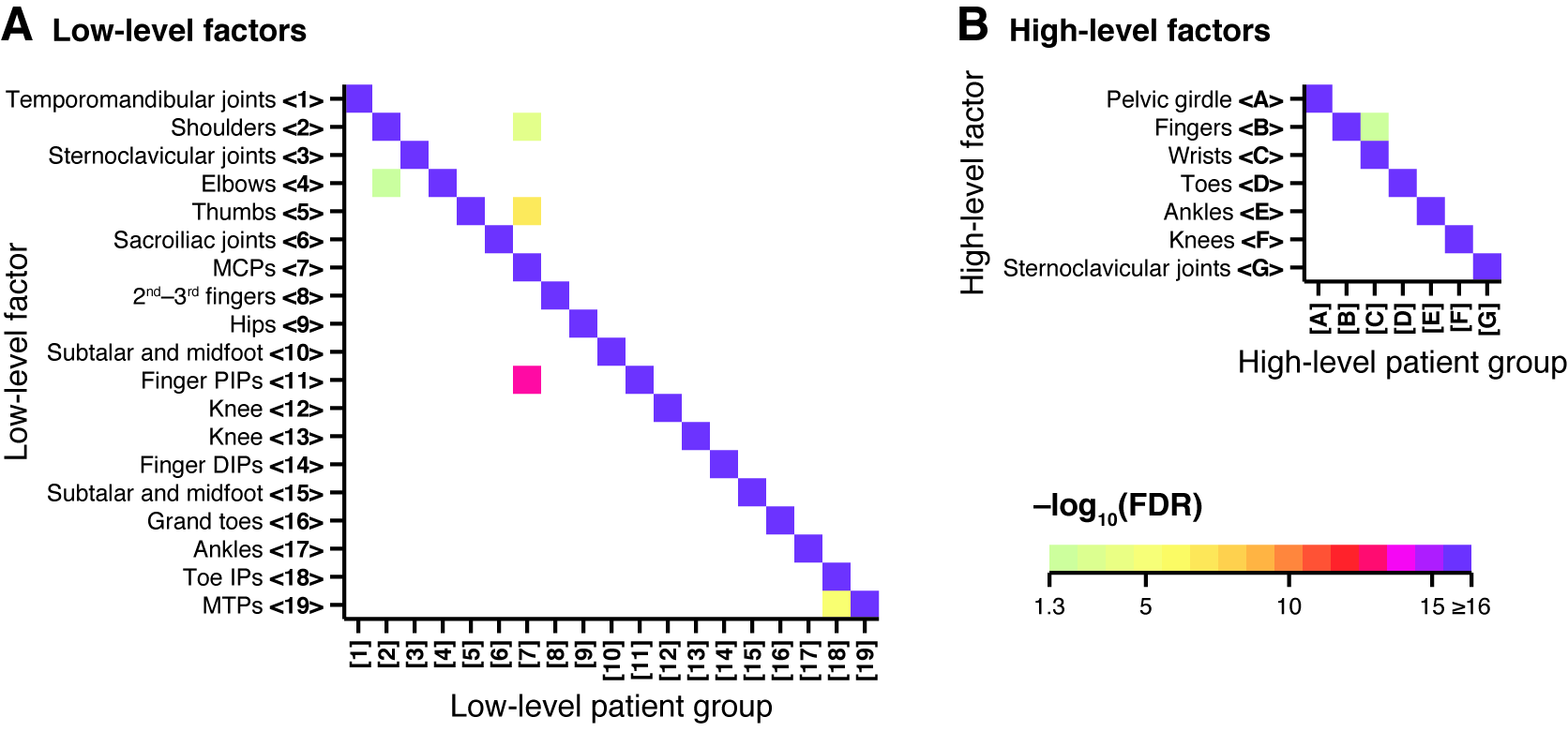

Supplement: S7 Fig — (A) Heat map of negative log-FDRs (colors; bottom-right legend) depicting the degree to which low-level patient groups (x-axis) associated with low-level factors (y-axis) based on z-tests. White denotes a negative log-FDR less than 1.3 (i.e., FDR < 0.05). (B) Same as panel A, but for high-level patient groups (x-axis) and high-level factors (y-axis). FDR, false discovery rate. (TIF) [file pmed.1002750.s008.tif]

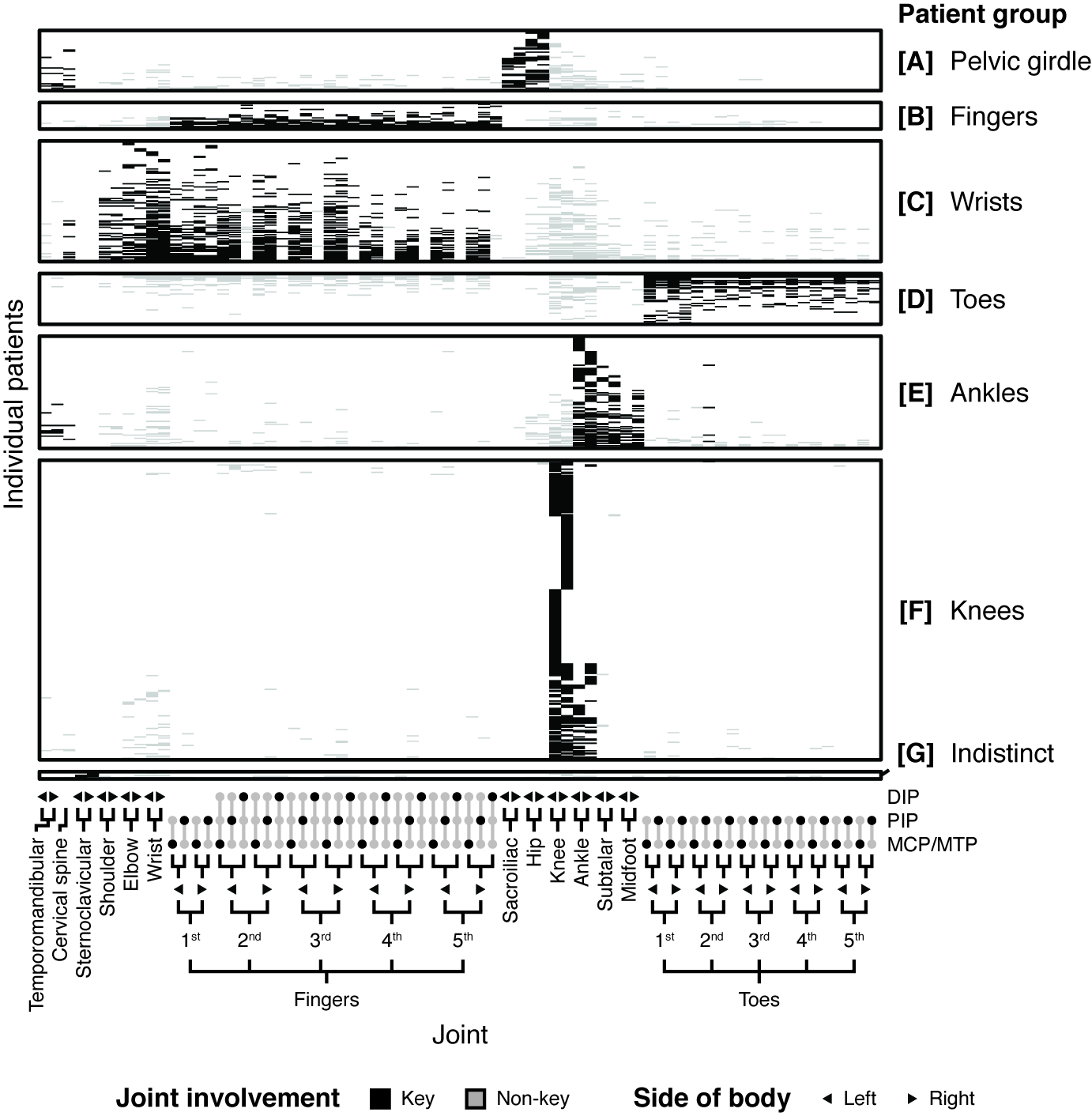

Supplement: S8 Fig — Heat map of individual joint involvements (x-axis) for each discovery cohort patient (y-axis), grouped by patient group (rows). Black cells indicate active joints that appear in high-level factors, or key joints, underlying patient groups (S6B Fig). Grey cells indicate other active joints (bottom-left legend). Left and right arrows (bottom-right legend) denote side of body. DIP, distal IP; IP, interphalangeal; MCP, metacarpophalangeal; MTP, metatarsophalangeal; PIP, proximal IP; TMJ, temporomandibular joint. (TIF) [file pmed.1002750.s009.tif]

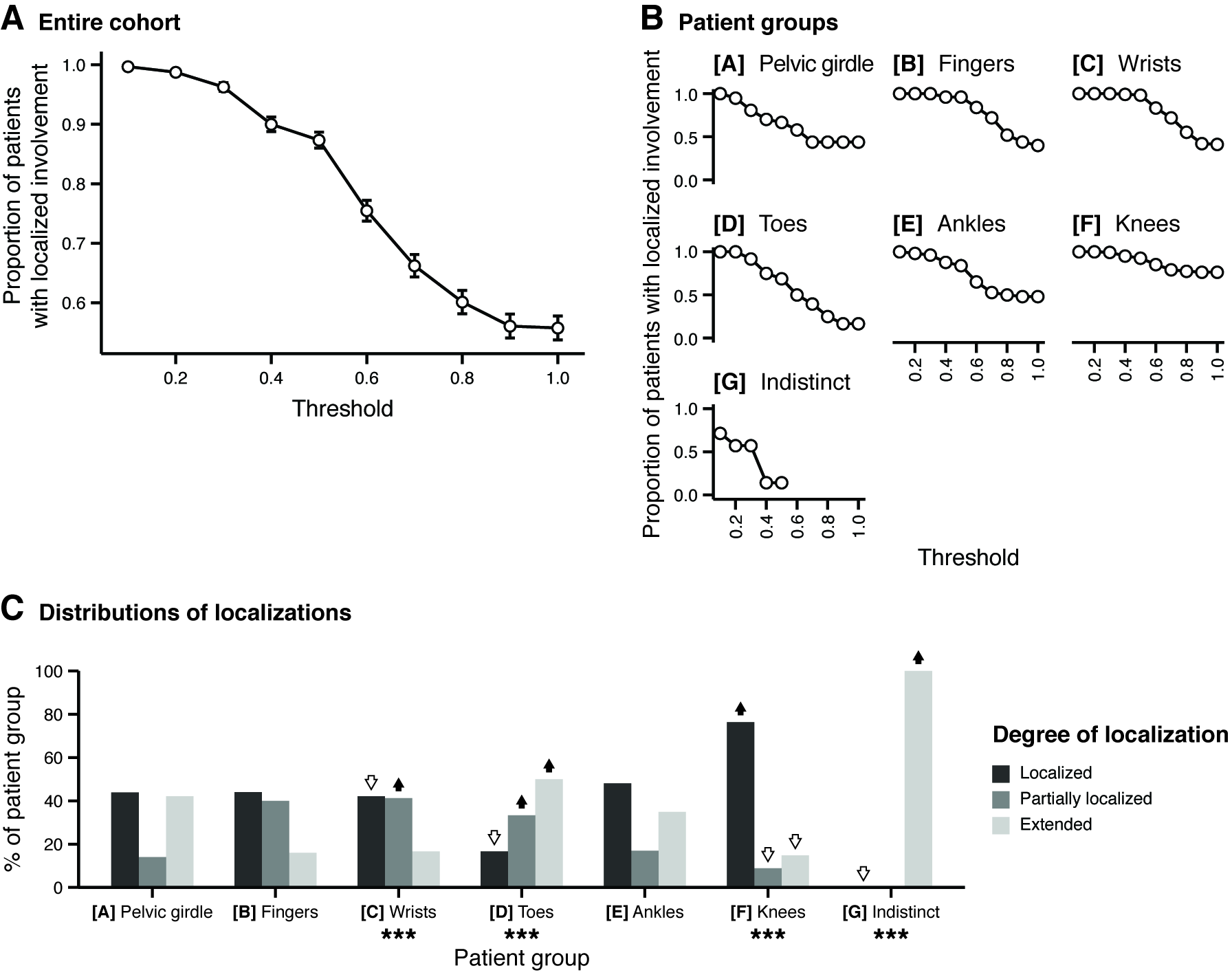

Supplement: S9 Fig — (A) At each given threshold (x-axis), the proportion of patients exceeding that threshold (y-axis). This threshold describes, for a given patient, the proportion of joints that are also key joints of that patient’s underlying high-level factor (S6B Fig). Error bars represent standard errors derived from 2,000 bootstraps. (B) Same as panel A but divided into patient groups (subpanels). (C) Bar graph of the percent of patients in each group with each localization (shades of grey; right legend). Up arrows denote enriched combinations of patient groups and localizations, and down arrows denote depleted or rarer combinations. ***P < 0.001 by χ2 test with Bonferroni correction for multiple hypothesis testing. (TIF) [file pmed.1002750.s010.tif]

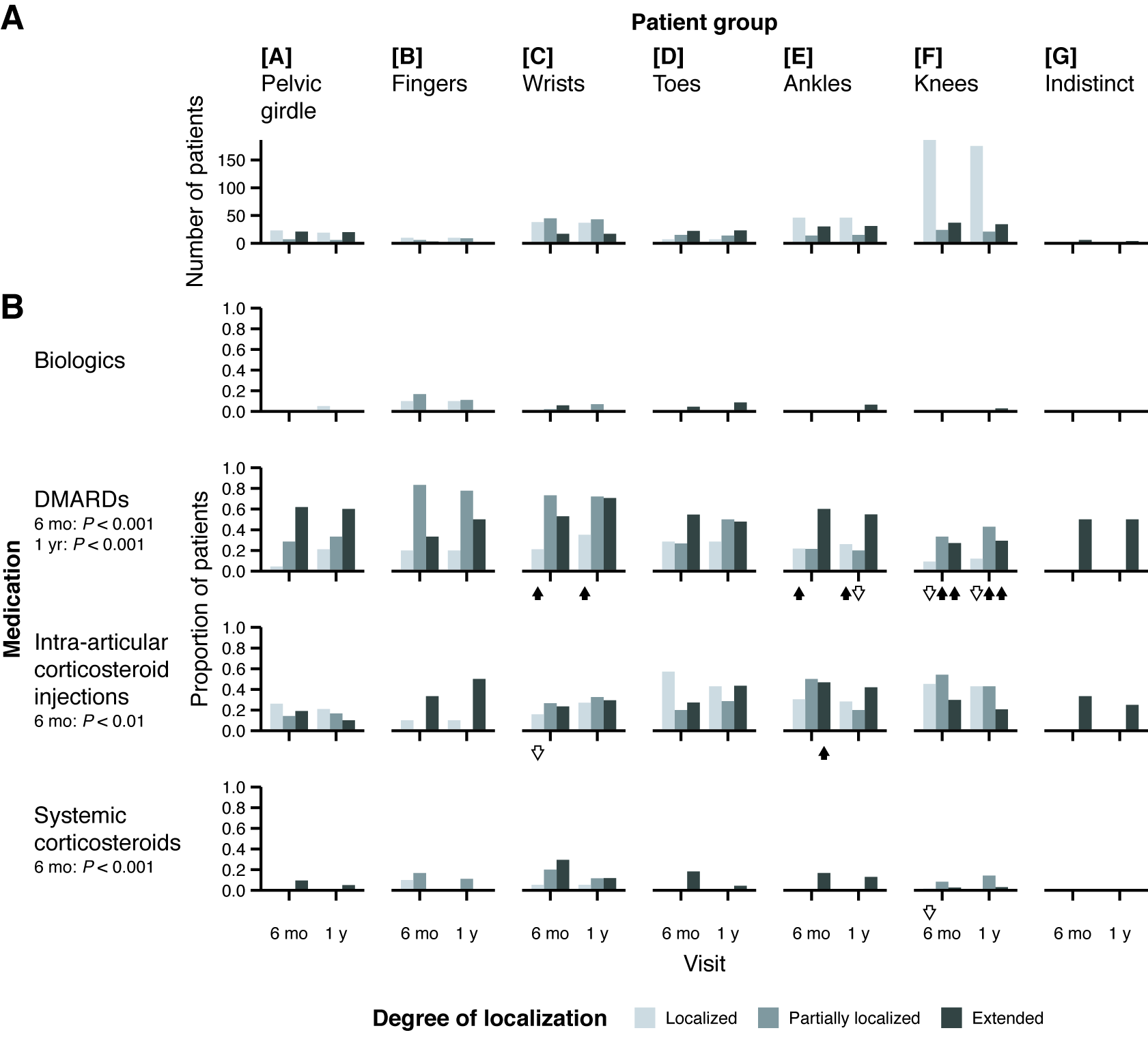

Supplement: S10 Fig — (A) Bar plots of the number of patients followed (y-axis) per patient group (columns) per visit (shades of grey; bottom legend). (B) Bar plots showing, for each treatment (rows), the proportion of patients (y-axes) per patient group (columns) and degree of localization (shades of grey; bottom legend) prescribed that treatment within six-month windows prior to six-month and one-year visits (x-axes). Up arrows denote, for each patient group, visit, and patient group, enriched localizations that occur more often than expected, and down arrows denote depleted localizations that occur less often than expected. (TIF) [file pmed.1002750.s011.tif]

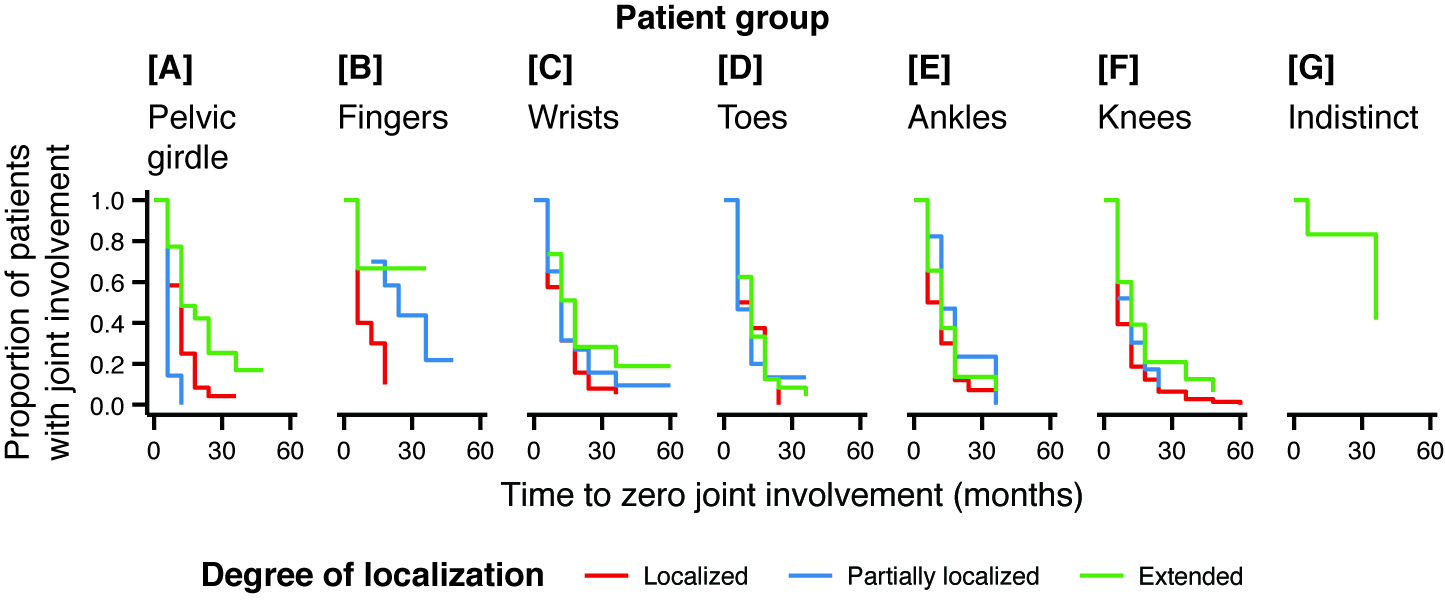

Supplement: S11 Fig — Time to zero curves for each patient group (columns) showing the proportion of patients continuing to have joint involvement (y-axes) after given visits after baseline (x-axes) and degree of localization (colors; bottom legend). (TIF) [file pmed.1002750.s012.tif]

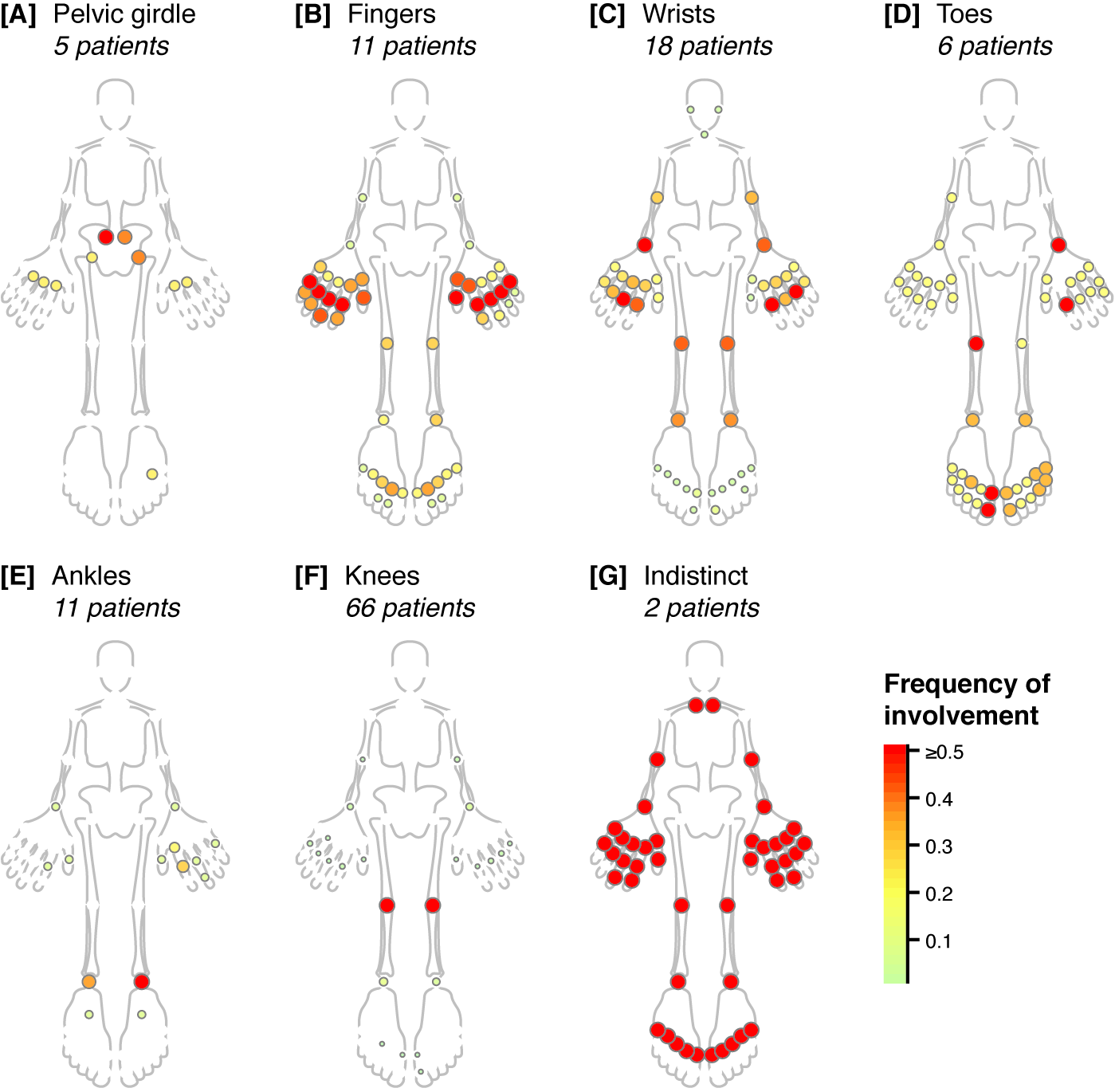

Supplement: S12 Fig — Homunculi of frequencies of involvement (colors; bottom-right legend) for individual joints (circles) for each projected patient group (panels) in the validation cohort. White denotes a probability of zero. See Fig 1 for the identity of each joint. (TIF) [file pmed.1002750.s013.tif]

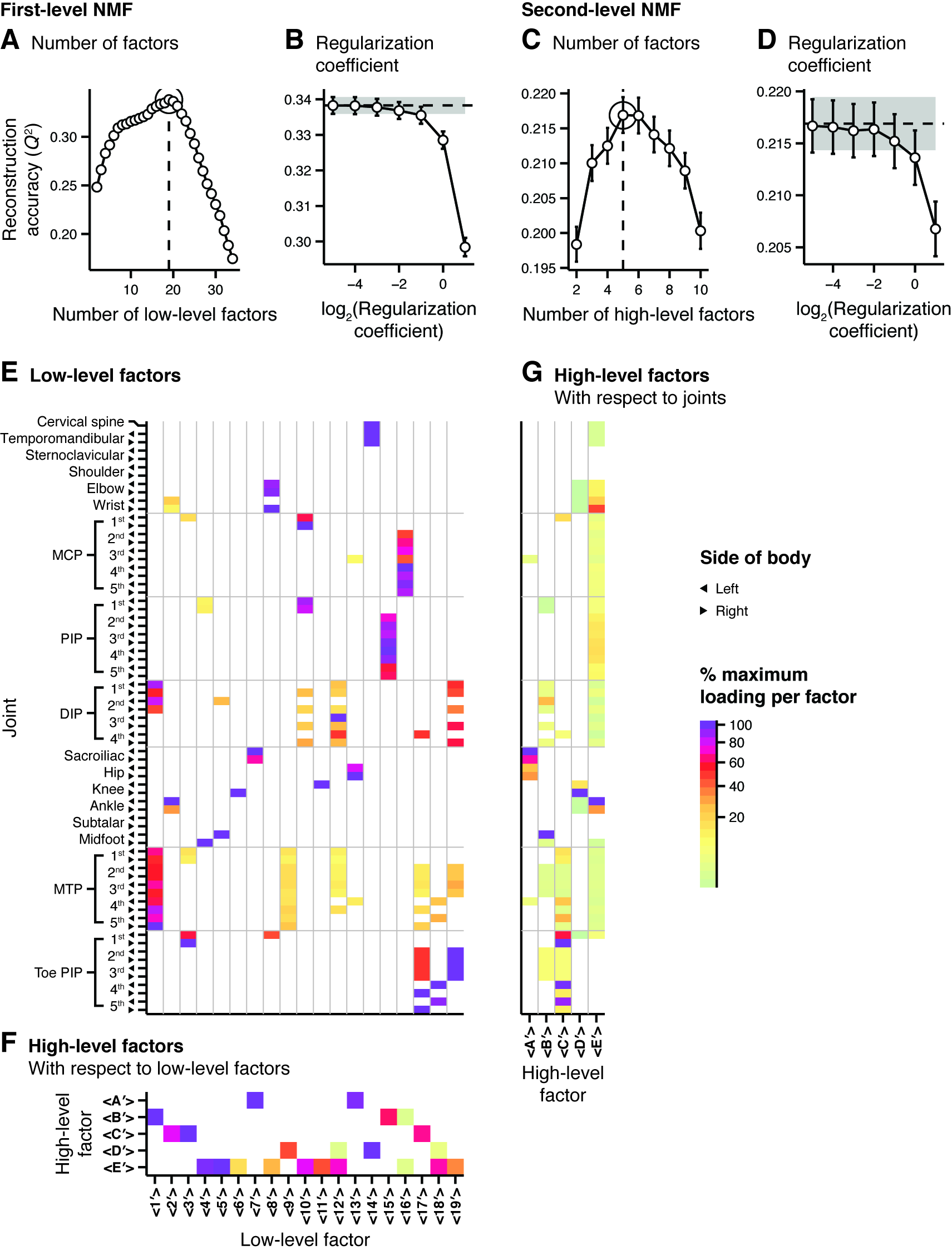

Supplement: S13 Fig — (A) Mean reconstruction accuracies (Q2; y-axis) of increasing numbers of low-level factors (x-axis). The highest Q2 is circled, and the corresponding number of factors is indicated by the dashed vertical line. Error bars represent 95% CIs. (B) Same as panel A, except for regularization coefficient (x-axis). The dashed horizontal line represents the mean Q2 when the regularization constant is zero, and the grey ribbon represents its standard deviation. (C) Same as panel A, except for high-level factors. (D) Same as panel B, except for high-level factors. (E) Heat map of contributions (colors; right lower legend) of joints (y-axis) to low-level factors (x-axis). White denotes zero contributions. Arrows denote side of body (right upper legend). (F) Heat map of contributions (colors; right lower legend) of low-level factors (x-axis) to high-level factors (y-axis). White denotes zero contributions. (G) Same as panel E, but for contributions of joints (y-axis) to high-level factors (x-axis). (TIF) [file pmed.1002750.s014.tif]

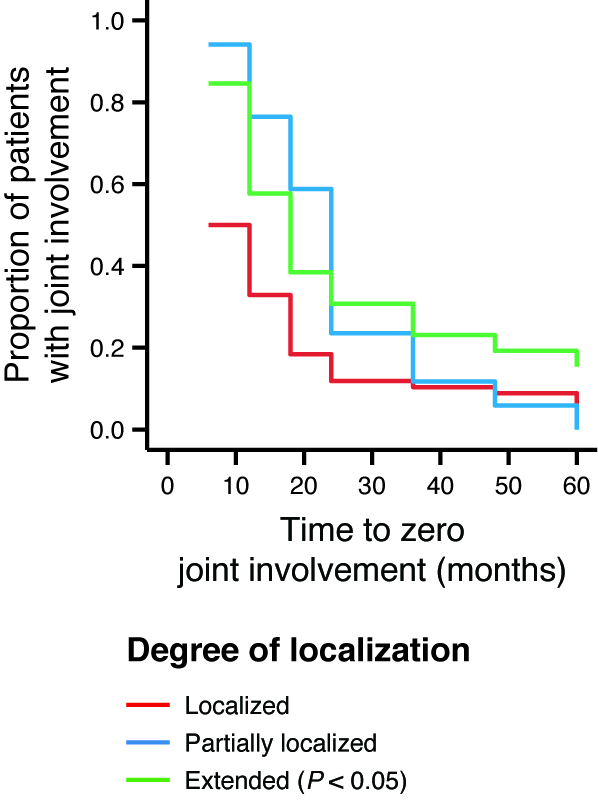

Supplement: S14 Fig — Time to zero curves, by degree of localization (colors; bottom legend), showing the proportion of patients continuing to have joint involvement (y-axes) after given visits after baseline (x-axes). (TIF) [file pmed.1002750.s015.tif]
