# Supplementary material for: Patterns of joint involvement in juvenile idiopathic arthritis and prediction of disease course: A prospective study with multilayer non-negative matrix factorization
Source: PLoS Med. 2019 Feb 26;16(2):e1002750. doi: 10.1371/journal.pmed.1002750 (PMC6390994; doi:10.1371/journal.pmed.1002750)
Supplement: S1 Table — ILAR, International League of Associations for Rheumatology. (DOCX) [file pmed.1002750.s016.docx]

| **Cohort** | **Analysis** | **Type** | ***Q*^2^** |
| --- | --- | --- | --- |
| Discovery | Factors | Low-level | 0.75 |
|  |  | High-level | 0.54 |
|  | Classifications | Patient groups | 0.31 |
|  |  | ILAR categories | 0.26 |
| Validation | Factors | Low-level, projected | 0.81 |
|  |  | Low-level, *de novo* | 0.84 |
|  |  | High-level, projected | 0.55 |
|  |  | High-level, *de novo* | 0.55 |
|  | Classifications | Patient groups | 0.48 |
|  |  | Patient groups, *de novo* | 0.35 |
|  |  | ILAR categories | 0.43 |
