# Supplementary material for: Patterns of joint involvement in juvenile idiopathic arthritis and prediction of disease course: A prospective study with multilayer non-negative matrix factorization
Source: PLoS Med. 2019 Feb 26;16(2):e1002750. doi: 10.1371/journal.pmed.1002750 (PMC6390994; doi:10.1371/journal.pmed.1002750)
Supplement: S2 Table — Standardized residuals ϵ generated from a χ2 test on a contingency table counting patients at the intersection of each patient group and ILAR subtype. *P < 0.05, **P < 0.01, ***P < 0.001. ILAR, International League of Associations for Rheumatology; RF, rheumatoid factor. (DOCX) [file pmed.1002750.s017.docx]

| **ILAR category** | **Patient group** | | | | | | |
| --- | --- | --- | --- | --- | --- | --- | --- |
|  | **[A]** | **[B]** | **[C]** | **[D]** | **[E]** | **[F]** | **[G]** |
| Systemic | –1.4 | 1.4 | 3.8*** | –1.3 | –0.80 | –1.8 | 1.7 |
| Oligoarthritis | –5.5*** | –3.7*** | –6.5*** | –4.3*** | –0.11 | 12.3*** | –1.8 |
| RF-negative polyarthritis | –0.21 | 2.8** | 6.1*** | 2.3* | 0.90 | –7.5*** | –0.29 |
| RF-positive polyarthritis | –0.31 | 0.56 | 5.0*** | 0.87 | –1.7 | –3.5*** | 2.1* |
| Psoriatic | 0.36 | 1.3 | 1.4 | 2.0* | –0.32 | –3.0** | –0.67 |
| Enthesitis-related arthritis | 8.1*** | –0.66 | –2.5** | 3.3*** | 1.7 | –5.7*** | 1.3 |
| Undifferentiated | 1.3 | 1.2 | –0.18 | –0.74 | –1.8 | –0.53 | 0.47 |
