# Supplementary material for: Patterns of joint involvement in juvenile idiopathic arthritis and prediction of disease course: A prospective study with multilayer non-negative matrix factorization
Source: PLoS Med. 2019 Feb 26;16(2):e1002750. doi: 10.1371/journal.pmed.1002750 (PMC6390994; doi:10.1371/journal.pmed.1002750)
Supplement: S3 Table — *P < 0.05, **P < 0.01, ***P < 0.001. (DOCX) [file pmed.1002750.s018.docx]

| **Patient group** | ***χ*^2^** | ***P*-value** | **Standardized residual by degree of localization** | | |
| --- | --- | --- | --- | --- | --- |
|  |  |  | **Localized** | **Partially localized** | **Extended** |
| **[A]** Pelvic girdle | 9.5 | 0.050 | –1.9 | –1.0 | 3.1 |
| **[B]** Fingers | 6.9 | 0.21 | –1.2 | 2.6* | –0.99 |
| **[C]** Wrists | 35 | <0.001*** | –3.0** | 5.9*** | –2.0 |
| **[D]** Toes | 31 | <0.001*** | –5.5*** | 2.4* | 4.1*** |
| **[E]** Ankles | 6.2 | 0.045* | –1.7 | –0.62 | 2.5* |
| **[F]** Knees | 48 | <0.001*** | 6.9*** | –4.5*** | –3.8*** |
| **[G]** Indistinct | 22 | <0.001*** | –3.0** | –1.3 | 4.6 |
