# Supplementary material for: Patterns of joint involvement in juvenile idiopathic arthritis and prediction of disease course: A prospective study with multilayer non-negative matrix factorization
Source: PLoS Med. 2019 Feb 26;16(2):e1002750. doi: 10.1371/journal.pmed.1002750 (PMC6390994; doi:10.1371/journal.pmed.1002750)
Supplement: S4 Table — Associations are enriched, or observed more often than expected, when the coefficient β > 0, and depleted when β < 0. DMARD, disease-modifying antirheumatic drug. (DOCX) [file pmed.1002750.s019.docx]

| **Medication** | **Visit** | **Patient group** | **Localization** | ***β*** | ***Z*** | ***P*-value** |
| --- | --- | --- | --- | --- | --- | --- |
| DMARDs | 6 months | **[C wrists]** | Localized | 0.97 | 2.1 | 0.039 |
|  |  | **[E ankles]** | Localized | 1.0 | 2.3 | 0.021 |
|  |  | **[F knees]** | Localized | –2.3 | –9.0 | <0.001 |
|  |  |  | Partial | 1.6 | 3.2 | 0.0014 |
|  |  |  | Extended | 1.3 | 2.9 | 0.0047 |
|  | 1 year | **[C wrists]** | Localized | 1.4 | 3.3 | <0.001 |
|  |  | **[E ankles]** | Localized | 0.95 | 2.3 | 0.020 |
|  |  |  | Partial | –2.0 | –2.3 | 0.020 |
|  |  | **[F knees]** | Localized | –2.0 | –8.6 | <0.001 |
|  |  |  | Partial | 1.7 | 3.4 | <0.001 |
|  |  |  | Extended | 1.1 | 2.5 | 0.012 |
| Joint injections | 6 months | **[C wrists]** | Localized | –1.5 | –3.2 | 0.0016 |
|  |  | **[E ankles]** | Extended | 1.4 | 2.2 | 0.029 |
| Systemic corticosteroids | 6 months | **[F knees]** | Localized | –4.1 | –7.1 | <0.001 |
