# Supplementary material for: Patterns of joint involvement in juvenile idiopathic arthritis and prediction of disease course: A prospective study with multilayer non-negative matrix factorization
Source: PLoS Med. 2019 Feb 26;16(2):e1002750. doi: 10.1371/journal.pmed.1002750 (PMC6390994; doi:10.1371/journal.pmed.1002750)
Supplement: S5 Table — *P < 0.05, **P < 0.01. CI, confidence interval. (DOCX) [file pmed.1002750.s020.docx]

| **Term** | **Hazard ratio** | | ***P*-value** |
| --- | --- | --- | --- |
|  | **Observed** | **95% CI** |  |
| **[A pelvic girdle]** | 0.89 | (0.62, 1.3) | 0.49 |
| **[B fingers]** | 0.64 | (0.38, 1.1) | 0.11 |
| **[C wrists]** | 0.88 | (0.66, 1.2) | 0.37 |
| **[D toes]** | 1.1 | (0.79, 1.6) | 0.50 |
| **[E ankles]** | 0.86 | (0.67, 1.1) | 0.24 |
| **[G indistinct]** | 0.25 | (0.060, 1.0) | 0.054 |
| Localization: partial | 0.70 | (0.56, 0.88) | 0.0018** |
| Localization: extended | 0.64 | (0.36, 0.88) | 0.0057** |
| Systemic arthritis | 0.84 | (0.49, 1.4) | 0.52 |
| RF-negative polyarthritis | 0.86 | (0.65, 1.1) | 0.31 |
| RF-positive polyarthritis | 0.42 | (0.21, 0.85) | 0.016* |
| Psoriatic arthritis | 1.0 | (0.68, 1.5) | 0.99 |
| Enthesitis-related arthritis | 0.97 | (0.71, 1.3) | 0.83 |
| Undifferentiated arthritis | 0.64 | (0.47, 0.88) | 0.0059** |
