# Supplementary material for: Patterns of joint involvement in juvenile idiopathic arthritis and prediction of disease course: A prospective study with multilayer non-negative matrix factorization
Source: PLoS Med. 2019 Feb 26;16(2):e1002750. doi: 10.1371/journal.pmed.1002750 (PMC6390994; doi:10.1371/journal.pmed.1002750)
Supplement: S1 Text — NMF, non-negative matrix factorization. (DOCX) [file pmed.1002750.s021.docx]

# S1 Text. Multilayer non-negative matrix factorization (NMF).

Prior to each level of multilayer NMF, we scaled joint involvements $\mathbf{X}^{\left( 1 \right)}$ and low-level patient factor scores $\mathbf{H}^{\left( 1 \right)}=\mathbf{X}^{\left( 2 \right)}$ to unit variance by dividing each variable by its standard deviation across patients, producing  ${\tilde{\mathbf{X}}}^{\left( 1 \right)}$ and ${\tilde{\mathbf{X}}}^{\left( 2 \right)}$ respectively.

We conducted NMF as implemented in the *scikit-learn* Python package, version 0.18 [1]. Non-negative double singular value decomposition [2] as implemented in *scikit-learn* was used to initialize NMF (init = nndsvd) and $L_{1}$ regularization was used to sparsify the resulting factors (l1_ratio = 1).

At each level $i$ of NMF, to determine the number of factors $k^{\left( i \right)}$, we fixed the regularization coefficient $\alpha^{\left( i \right)}$ to zero and conducted two rounds of three-fold bi-cross-validation (BiCV) with simple residuals [3]. This procedure withheld one third of patients and measurements in the joint involvement data as *validation data*. With the remaining data or *training data*, we calculated basis/loading and coefficient/score matrices and reconstructed validation data from these matrices. We evaluated reconstruction accuracy using *Q*^2^ [4], which correlates the original validation data with their reconstructions, and selected the number of factors $k$ with the highest mean *Q*^2^.

To determine the regularization coefficient $\alpha^{\left( i \right)}$, we fixed the number of factors to $k^{\left( i \right)}$ and conducted BiCV with $\boldsymbol{\alpha}=2^{x},x\in\left\{ -5,-4,\ldots,1 \right\}$. We expected *Q*^2^ to be highest at $\alpha^{\left( i \right)}=0$ as fewer basis matrix entries are coerced to zero due to regularization. Therefore, we calculated a *Q*^2^ threshold equal to the mean *Q*^2^ at $\alpha^{\left( i \right)}=0$ minus its standard error of the mean. We selected the highest $\alpha^{\left( i \right)}$ whose mean *Q*^2^ was greater than that threshold [5] and conducted NMF on the full joint involvement data with the chosen number of factors $k$ and regularization coefficient $\alpha$.

After conducting first-level NMF, we conducted second-level NMF on low-level patient factor scores ${\tilde{\mathbf{H}}}^{\left( 1 \right)}={\tilde{\mathbf{X}}}^{\left( 2 \right)}$ as per the first-level analysis above, producing high-level factors.

Overlap between factors occurs when individual joints appear in multiple factors. To further reduce overlap, we sparsified factors. For each factor, we defined a threshold equal to half of the maximum entry in that factor’s basis vector and zeroed basis matrix entries below this threshold. We then rescaled basis matrix factors such that their *L*_2_ norms matched those prior to sparsification.

1. Pedregosa F, Varoquaux G, Gramfort A, Michel V, Thirion B, Grisel O, et al. Scikit-learn: Machine Learning in Python. The Journal of Machine Learning Research. JMLR.org; 2011;12: 2825–2830.

2. Boutsidis C, Gallopoulos E. SVD based initialization: A head start for nonnegative matrix factorization. Pattern Recognition. 2008;41: 1350–1362. doi:10.1016/j.patcog.2007.09.010

3. Owen AB, Perry PO. Bi-cross-validation of the SVD and the nonnegative matrix factorization. The Annals of Applied Statistics. 2009.

4. Stacklies W, Redestig H, Scholz M, Walther D, Selbig J. pcaMethods--a bioconductor package providing PCA methods for incomplete data. Bioinformatics. 2007;23: 1164–1167. doi:10.1093/bioinformatics/btm069

5. Hastie T, Tibshirani R, Friedman J. The Elements of Statistical Learning. 2nd ed. 2009.
