# Supplementary material for: Patterns of joint involvement in juvenile idiopathic arthritis and prediction of disease course: A prospective study with multilayer non-negative matrix factorization
Source: PLoS Med. 2019 Feb 26;16(2):e1002750. doi: 10.1371/journal.pmed.1002750 (PMC6390994; doi:10.1371/journal.pmed.1002750)
Supplement: S2 Text — (DOCX) [file pmed.1002750.s022.docx]

# S2 Text. Localizations.

To further stratify patients by the degree of localization of their active joints, we conducted a bootstrap analysis. In this analysis, we calculated a threshold $x_{\text{localized}}$ based on the proportions of active joints $\mathbf{p}$ that are also key joints in high-level factors underlying patient group assignments. For example, if $x_{\mathrm{localized}}=1$, a patient with only knee arthritis would have 100% of their active joints appearing in **<F knees>** and therefore would be *localized* with respect to **<F>** (see Results). We considered patients with $\mathbf{p}\geq x_{\text{localized}}$ as having *localized* involvement. The number of such patients is $n_{x_{\mathrm{localized}}}$. We chose the highest threshold $x_{\text{localized}}\in(0, 0.1, \ldots, 1)$ that satisfied

$$\mu\left( n_{x_{\text{localized}}} \right)<\mu\left( n_{x_{\text{localized}}=1} \right)-SEM(n_{x_{\text{localized}}=1})$$

where $\mu(\ldots)$ is the bootstrap mean and $SEM\left( \ldots\right)$ is the standard error of the mean.

We defined an additional category, *partially localized* involvement, by determining which threshold $x_{\mathrm{partial}}$ had the lowest slope $m$ as calculated by $m=\frac{p_{x+0.1}-p_{x-0.1}}{0.2}$. We classified patients with $\mathbf{p}<x_{\mathrm{partial}}$ as having *extended* involvement, involvement of joints beyond those defining their underlying high-level factors.
