# Supplementary material for: Patterns of joint involvement in juvenile idiopathic arthritis and prediction of disease course: A prospective study with multilayer non-negative matrix factorization
Source: PLoS Med. 2019 Feb 26;16(2):e1002750. doi: 10.1371/journal.pmed.1002750 (PMC6390994; doi:10.1371/journal.pmed.1002750)
Supplement: S3 Text — (DOCX) [file pmed.1002750.s023.docx]

# S3 Text. Logical patterns of joint involvement.

First-level non-negative matrix factorization (NMF) identified 19 low-level factors that grouped joints along the horizontal body axis. 19 low-level factors had a mean bi-cross-validation (BiCV) reconstruction accuracy, or *Q*^2^, of 0.41 with respect to the original joint involvement data when the regularization constant *α* was fixed to zero (S3 Fig [A]). BiCV on *α* with *k* = 19 chose *α* = 0.50, whose mean *Q*^2^ = 0.41 was not below the threshold  ${\bar{Q^{2}}}_{\alpha=0}-SE_{\alpha=0}=0.41-0.00072=0.41$ (S3 Fig [B]). Sparsifying factors (see S1 Text) maintained relationships between patients on low-level factors (S4 Fig [A]). Across most factors, we found little deviation from the diagonal lines of best fit, which intersected both the sparsified and unsparsified score axes near (0, 0) and exhibited slopes of one. The resulting 19 intermediate factors, **<1–19>**, described themes of joint involvement described in the main body of this paper when visualized as a heat map of contributions of joints to low-level factors (S5 Fig [A] and S6 Fig [A]).

Second-level NMF identified seven high-level patterns of broader joint involvement from the granular low-level patterns. Seven factors had a mean BiCV *Q*^2^ of 0.30 with respect to the patient low-level factor scores when *α* = 0 (S3 Fig [C]) and *α* = 0.50 had a mean *Q*^2^ of 0.30 (S3 Fig [D]). Sparsifying factors maintained relationships between patients on high-level factors (S4 Fig [B]) as with the low-level factors.

Factors robustly represented joint involvement data. Joint involvement data reconstructed from low-level factors, high-level factors, and patient groups had *Q*^2^ values of 0.75, 0.54, and 0.31 respectively as measured over the entire discovery data (S3 Table). These values compared favourably against ILAR categories, with a *Q*^2^ of 0.26. The seven high-level factors, **<A–G>**, grouped low-level factors by localization around the body, which became apparent in heat maps of unsparsified and sparsified contributions of low-level factors to these factors (S5 Fig [BC] and S6 Fig [BC]). For example, metacarpophalangeal joints in **<7>** and proximal interphalangeal joints in **<11>** merged into one factor, **<C wrists>**.
