# Supplementary material for: Patterns of joint involvement in juvenile idiopathic arthritis and prediction of disease course: A prospective study with multilayer non-negative matrix factorization
Source: PLoS Med. 2019 Feb 26;16(2):e1002750. doi: 10.1371/journal.pmed.1002750 (PMC6390994; doi:10.1371/journal.pmed.1002750)
Supplement: S4 Text — (DOCX) [file pmed.1002750.s024.docx]

# S4 Text. Sub-categorizing patients by degree of joint localization.

S9 Fig (A) shows the proportion of patients with localized joint involvement based on the proportion of active joints matching those in the high-level factors. A threshold of 0.9, or 90% of joints appearing as such, did not result in more patients with localized involvement than a threshold of 1.0. The largest negative slope in the resulting curve was at a threshold of 0.6 (60%). Therefore, we assigned patients with 90% of their active joints matching their underlying high-level factors as having *localized* involvement, patients with 60% to 90% as having *partially* localized involvement, and less than 60% as having *extended* involvement.
