# Supplementary material for: Patterns of joint involvement in juvenile idiopathic arthritis and prediction of disease course: A prospective study with multilayer non-negative matrix factorization
Source: PLoS Med. 2019 Feb 26;16(2):e1002750. doi: 10.1371/journal.pmed.1002750 (PMC6390994; doi:10.1371/journal.pmed.1002750)
Supplement: S5 Text — (DOCX) [file pmed.1002750.s025.docx]

# S5 Text. *De novo* validation.

First-level non-negative matrix factorization (NMF) identified 19 low-level factors whose mean bi-cross-validation (BiCV) reconstruction accuracy, *Q*^2^, was 0.41 with respect to the original joint involvement data when the regularization constant *α* was fixed to zero (S13 Fig [A]). BiCV on *α* with *k* = 19 chose *α* = 0.25, whose mean *Q*^2^ = 0.43 was not below the threshold  ${\bar{Q^{2}}}_{\alpha=0}-SE_{\alpha=0}=0.43-0.0018=0.43$ (S13 Fig [B]). S13 Fig (E) visualizes the contributions of joints to low-level factors.

Second-level NMF identified five high-level patterns of joint involvement from the low-level factors. These factors had a mean BiCV *Q*^2^ of 0.31 with respect to low-level factor scores when *α* = 0 (S13 Fig [C]). *α* = 0.50 had a mean *Q*^2^ of 0.31 (S13 Fig [D]). S13 Fig (F) shows the contributions of low-level factors to high-level factors and S13 Fig (G) the contributions of joints to high-level factors.
